# Estimating the cost of achieving basic water, sanitation, hygiene, and waste management services in public health-care facilities in the 46 UN designated least-developed countries: a modelling study

**DOI:** 10.1016/S2214-109X(22)00099-7

**Published:** 2022-04-06

**Authors:** Michael Chaitkin, Samantha McCormick, Jorge Alvarez-Sala Torreano, Irene Amongin, Silvia Gaya, Odd N Hanssen, Richard Johnston, Tom Slaymaker, Claire Chase, Guy Hutton, Maggie Montgomery

**Affiliations:** a Englewood, CO, USA; b Washington, DC, USA; c Division of Water, Sanitation and Hygiene, UNICEF, New York, NY, USA; d Division of Data, Analytics, Planning and Monitoring, UNICEF, New York, NY, USA; e Center for Economic and Policy Research, Washington, DC, USA; f Water, Sanitation, Hygiene and Health Unit, WHO, Geneva, Switzerland; g Water Global Practice, The World Bank, Washington, DC, USA

## Abstract

**Background:**

An alarming number of public health-care facilities in low-income and middle-income countries lack basic water, sanitation, hygiene (WASH), and waste management services. This study estimates the costs of achieving full coverage of basic WASH and waste services in existing public health facilities in the 46 UN designated least-developed countries (LDCs).

**Methods:**

In this modelling study, in-need facilities were quantified by combining published counts of public facilities with estimated basic WASH and waste service coverage. Country-specific per-facility capital and recurrent costs to deliver basic services were collected via survey of country WASH experts and officials between Sept 24 and Dec 24, 2020. Baseline cost estimates were modelled and discounted by 5% per year. Key assumptions were adjusted to produce lower and upper estimates, including adjusting the discount rate to 8% and 3% per year, respectively.

**Findings:**

An estimated US$6·5 billion to $9·6 billion from 2021 to 2030 is needed to achieve full coverage of basic WASH and waste services in public health facilities in LDCs. Capital costs are $2·9 billion to $4·8 billion and recurrent costs are $3·6 billion to $4·8 billion over this time period. A mean of $0·24–0·40 per capita in capital investment is needed each year, and annual operations and maintenance costs are expected to increase from $0·10 in 2021 to $0·39–0·60 in 2030. Waste management accounts for the greatest share of costs, requiring $3·7 billion (46·6% of the total) in the baseline estimates, followed by $1·8 billion (23·1%) for sanitation, $1·5 billion (19·5%) for water, and $845 million (10·7%) for hygiene. Needs are greatest for non-hospital facilities ($7·4 billion [94%] of $7·9 billion) and for facilities in rural areas ($5·3 billion [68%]).

**Interpretation:**

Investment will need to increase to reach full coverage of basic WASH and waste services in public health facilities. Financial needs are modest compared with current overall health and WASH spending, and better service coverage will yield substantial health benefits. To sustain services and prevent degradation and early replacement, countries will need to routinely budget for operations and maintenance of WASH and waste management assets.

**Funding:**

WHO (including underlying grants from the governments of Japan, the Netherlands, and the UK), World Bank (including an underlying grant from the Global Water Security and Sanitation Partnership), and UNICEF.

**Translations:**

For the Arabic, French and Portuguese translations of the abstract see Supplementary Materials section.

## Introduction

At the 2019 World Health Assembly, all 194 WHO member states resolved to ensure that every health-care facility in the world has adequate water, sanitation, and hygiene (WASH) services, waste management, and environmental cleaning practices.^1^ Ministries of Health committed to set, implement, and regularly monitor standards, as well as to empower the health workforce to improve WASH and waste management practices. These commitments echoed the UN Secretary-General's 2018 call to action^2^ and the growing global collaboration on WASH in health-care facilities co-led by WHO and UNICEF.^3^ Member states recognised that the lack of WASH and waste services and behaviours forestall progress towards the Sustainable Development Goals (SDGs), especially the attainment of healthy lives and wellbeing (goal 3) and water and sanitation for all (goal 6). This collective action came amidst intensifying efforts to track access to WASH and waste services in health-care settings, aided by global indicators and service levels defined by the WHO/UNICEF Joint Monitoring Programme (JMP) for Water Supply, Sanitation, and Hygiene,^4^ setting of a global target for all health-care facilities to have basic WASH and waste services by 2030 as input to the SDGs agenda,^3^ and publication of global coverage estimates for the first time.^5^ Despite progress, in 2019 an estimated quarter of health facilities still did not have basic water services, a tenth had no sanitation services, over one third did not have hand hygiene at points of care, and three out of ten failed to safely segregate waste.^6^

**Research in context**

**Evidence before this study**

We consulted widely within WHO (including with staff in regional and country offices and headquarters teams focused on health financing, health workforce, health systems, and health emergencies), with representatives of relevant partner agencies (including World Bank; the Global Fund to Fight AIDS, Tuberculosis, and Malaria; Water Aid; and World Vision), and with other experts working on water, sanitation, hygiene (WASH) and waste management in health-care facilities globally. All indicated that no comprehensive costing for WASH and waste services in health-care facilities had been previously conducted for the UN designated least-developed countries (LDCs). We also searched PubMed, Jisc Library Hub Discover, and Google Scholar using the terms “water”, “sanitation”, “hygiene”, “health care waste”, “costs”, and “costing” for articles published in English until Aug 31, 2020, and did not find any global or LDC-focused studies. Related resource needs have previously been estimated for achieving the health-related and WASH-related Sustainable Development Goals (SDGs). In 2016, the World Bank estimated that $28 billion (US$ 2015) was needed annually between 2015 and 2030 to provide universal access to basic WASH services (SDG 6: safe water and sanitation for all) in 140 low-income and middle-income countries (LMICs). These estimates, and the update for sanitation published by UNICEF in 2020, addressed the needs of households but not institutions such as schools or health-care facilities. In 2017, WHO estimated that an additional $274 billion (US$ 2014) per year, between 2016 and 2030, would allow 67 LMICs to achieve SDG 3 (healthy lives and wellbeing). This estimate only partially accounted for WASH and waste management needs in health-care facilities, for which WHO did not report specific findings. A 2021 systematic review by Anderson and colleagues found only 36 studies on environmental health service costs in health-care facilities in LMICs; of these, only three studies were conducted during the SDGs era in one of the currently UN designated LDCs (Rwanda, Malawi, and Zambia), and none presented national resource needs estimates. At the end of 2020, a provisional cost estimate for achieving full coverage of WASH and waste services in facilities in the LDCs was included in WHO and UNICEF's global progress report on WASH in health-care facilities but without a discussion of methodological details. This study updates and substantiates that estimate.

**Added value of this study**

To our knowledge, this is the first study to quantify the costs of achieving global targets specifically for WASH and waste services in health-care settings. Given the poor state of WASH and waste services in LDCs, substantial investment will be needed to achieve coverage in all existing public health-care facilities by 2030. We estimated the total capital and recurrent costs necessary to provide basic WASH and waste management services. Our analysis benefited from a new set of per-facility cost data rapidly collected by UNICEF in late 2020 via a survey of WASH experts and government officials working in nearly 60 LMICs.

**Implications of all the available evidence**

We found that achieving full coverage of basic WASH and waste services in the LDCs' existing public health-care facilities will require substantial investment, although the needs are modest when compared with prevailing government and donor resource flows for health and WASH. Waste management accounts for nearly half the resource needs, with lesser shares for sanitation, water, and hygiene. Most additional spending is required in non-hospital facilities and in facilities in rural areas, meaning efforts to meet WASH and waste needs in public health-care facilities will contribute to the equity-centred and primary care-centred principles of the post-2015 development agenda. Our estimates can inform resource mobilisation, planning, and prioritisation efforts within global and national public health and WASH communities. The estimates can also help to stimulate policy dialogue regarding the distribution of financial and operational responsibilities for environmental health services across sectors, administrative levels of government, and the private sector.

These deficits contribute to the health and economic harms wrought by poor-quality health care. In low-income and middle-income countries, more deaths occur due to low service quality than from lack of access to care,^7^ and lost productivity from poor-quality care costs an estimated US$1·4 trillion to $1·6 trillion each year.^8^ Inadequate WASH and waste management in health-care facilities increases the likelihood of health care–associated infections^9^ and contributes to antimicrobial resistance.^10^ The global spread of SARS-CoV-2, the virus that causes COVID-19, draws further attention to these risks given the importance of WASH and waste services for effective infection prevention and control, health worker safety, and the continuity of essential services.^11^

Achieving full coverage of basic WASH and waste services in health facilities by 2030 will require considerable efforts to build, rehabilitate, operate, and maintain infrastructure, but the costs of doing so have not been estimated. Existing global resource-needs estimates for reaching the SDG targets for WASH focus on household access, not health facilities or other institutional settings.^12^, ^13^ Global price tags for the health SDG targets^14^ and primary health care^15^ only partially account for health facilities' WASH and waste management needs, and they do not include the facilities on which the poorest often rely, such as clinics and health posts. Global cost projections for effective national COVID-19 responses^16^ and vaccine roll-outs^17^ incorporate some considerations of water, hygiene, and waste management in health facilities, but they are based on emergency response strategies rather than sustainable, long-term solutions, and they do not include sanitation. Few studies have estimated high-quality facility-level or national-level costs on which global estimates could be based.^18^

To inform global resource mobilisation efforts for critical health infrastructure needs, this study estimates the cost of achieving full coverage of basic WASH and waste services in existing public health-care facilities in the UN designated least developed countries (LDCs) by 2030. This study updates and substantiates a preliminary estimate of $3·4 billion that WHO and UNICEF published in late 2020 within a broader global progress report (appendix 4 p 3).^6^

## Methods

### Study design

In this modelling study, we estimate the financial costs of achieving full coverage by 2030 of basic WASH and waste management services in existing public health-care facilities in the 46 LDCs, home to 1·1 billion people (appendix 4 p 4). The focus on LDCs reflects both a scarcity of coverage data in middle-income and high-income countries and a desire to prioritise attention and investment to the countries with the greatest needs. In LDCs, half of all facilities did not have basic water services, nearly two-thirds did not have basic sanitation, a quarter were without basic hygiene at points of care, and 70% did not adequately manage waste in 2019.^6^

Costs are estimated from the provider perspective, namely the public sector organisations responsible for health and infrastructure. The analysis does not distinguish among current financing sources, which can include government, the private sector, donors, and others. The estimated costs are additional to what is already being spent; the analysis therefore assumes that countries will sustain service coverage where it already exists. The definitions used for global monitoring of basic service levels for WASH in health-care facilities (developed by the JMP-convened Global Task Team for Monitoring WASH in Health Care Facilities)^4^ are shown in the panel.

Panel Definitions of basic service levels in health-care facilities**The global monitoring definitions were developed by the Global Task Team for Monitoring WASH in Health Care Facilities in the Sustainable Development Goals Era, convened by the WHO/UNICEF Joint Monitoring Programme for Water Supply, Sanitation, and Hygiene under the auspices of the Global Action Plan on WASH in Health Care Facilities. More information can be found from WHO and UNICEF.4

**Water**

Water is available from an improved source on the premises.

**Sanitation**

Improved sanitation facilities are usable, with at least one toilet dedicated for staff, at least one sex-separated toilet with menstrual hygiene facilities, and at least one toilet accessible for people with limited mobility.

**Hygiene**

Functional hand hygiene facilities (with water and soap or alcohol-based hand rub, or both) are available at points of care and within 5 m of toilets.

**Waste management**

Waste is safely segregated into at least three bins, and sharps and infectious waste are treated and disposed of safely.

**Environmental cleaning** †

Basic protocols for cleaning are available, and staff with cleaning responsibilities have all received training.

WASH=water, sanitation, and hygiene.

### Per-facility costs

Estimation of per-facility costs relied on unpublished data collected between Sept 24 and Dec 24, 2020 (appendix 4 pp 5–12). Experts in UNICEF's country offices were surveyed for information regarding the average costs per facility of improving from an absence of WASH and waste services to meeting the JMP's monitoring definitions for basic services. Data on upfront investments (capital costs) and annual operations and maintenance (recurrent costs) were collected for WASH and waste management services across different facility types (hospitals and non-hospitals) and settings (urban and rural). No additional guidance was given to respondents regarding facility size; rather, it was assumed they accounted for variation in their submissions. In most countries, UNICEF personnel consulted with health ministries to complete the survey. Respondents were instructed to provide average costs, expressed in 2020 US$, based on standard technologies available in their countries. By Dec 31, 2020, a database constructed from their responses contained at least some cost information for 40 of the 46 LDCs (home to 95% of the LDC population). Regional and all-LDC median costs were used when values were missing (appendix 4 pp 5–12).

### Identifying and characterising facilities

Extensive internet searches yielded primary and secondary data on public sector facility counts from national governments and international agencies (appendix 4 pp 13–19). Public primary, secondary, tertiary, and more advanced or specialised facilities were identified, with private facilities excluded unless managed as part of the public system. In line with how the per-facility cost survey differentiated facilities based on type and setting, the identified facilities were sorted into four profiles: urban hospitals, urban non-hospitals, rural hospitals, and rural non-hospitals (appendix 4 pp 13–19). Hospitals included all tertiary and more advanced or specialised facilities, while non-hospitals were defined broadly to include most fixed-location establishments not classified as hospitals. The inclusion of all permanent primary facilities contrasts with other global price tags for health, which include health centres but exclude lower-level clinics and health posts.^14^, ^15^, ^16^

### Quantifying needs

For each service, a facility in need was defined as one that did not already meet or exceed the basic service level. Coverage data were retrieved from the JMP database for 2019, the most recent year available. Each country's own estimates were applied whenever possible; otherwise, the JMP's all-LDC estimates were applied. Rules were developed to match stratified coverage estimates to the four facility profiles, based either on facility attributes (type and location) or, when there were no country estimates for the preferred strata, on correlation analyses used to rank the alternatives (appendix 4 pp 20–23). Given variation among sub-standard facilities (ranging from a complete absence of services to requiring only minor improvements), more detailed needs categories were defined for each service, leveraging JMP data for indicators such as the share of facilities that had an improved, on-premises water source but still fell short of the basic service level (all needs categories are summarised in appendix 4 [pp 20–23] with corresponding indicators and cost assumptions).

### Assigning water and sanitation services

In-need facilities were assigned a water or sanitation technology to align with the per-facility cost data. For water, per-facility costs were available for connecting to piped networks or exploiting on-premises water sources, such as boreholes or rainwater collection systems. For sanitation, per-facility costs were available for sanitation facilities connected either to a sewer or to a septic tank. By contrast, only one service option each was reflected in the per-facility cost data for hygiene and waste management.

Data on the availability of networked water and sanitation services (ie, piped water and sewerage-based sanitation) came from the comments section of the per-facility cost survey and the JMP country files, which consolidate findings from nationally representative health facility assessments. A technology was considered unavailable in a country if so indicated by the survey response, except in rare instances when the JMP data indicated coverage of at least 10%, in which case the JMP estimate was used. Where data for health-care facilities were not available, household data from the JMP were used as proxies. Similar to the method for quantifying needs, rules were developed to match stratified service availability estimates to the four facility profiles (appendix 4 pp 24–25). For each country, these data determined what share of in-need facilities were assigned a networked service, with the remainder assigned to on-premises water sources and sanitation systems.

### Model specifications

Data were combined in an Excel-based model that computed the aggregate capital and recurrent costs required to progress from current to full coverage of basic WASH and waste services in all LDCs by 2030, the year by which all facilities are meant to have basic services.^3^ The model assumed a linear scale-up of investment, such that capital costs were spread evenly across the ten-year period ending in 2030, with corresponding increases to annual recurrent costs. Per-capita estimates were based on country populations from the UN's medium variant population projections for 2021 to 2030.^19^ Replacement costs were incorporated for services whose assets were expected to expire before 2030, including hygiene in facilities with non-piped water sources and incinerators in non-hospitals. Replacement costs were incurred entirely within the year following asset expiration (appendix 4 pp 26–27).

Future costs were discounted to present value terms at a 5% annual rate, which was the most commonly applied rate found in a 2018 review of national practices,^20^ and which falls within the range of prominent methodological guidance (appendix 4 pp 28–29). The model's estimates for all LDCs were computed by aggregating country-level costs; however, data confidentiality agreements prevent the presentation of country-specific findings.

### Sensitivity analysis

To address the uncertainty in identifying country-specific coverage levels and per-facility costs, and in recognition that investment decisions are made in diverse and evolving contexts, lower and upper estimates were also generated by varying key model assumptions. While facilities requiring partial investment for water and sanitation were assumed to need 50% of the full per-facility capital costs in the baseline estimates, they were assigned 15% of those costs for the lower estimate and 85% of those costs for the upper estimates. Additionally, the discount rate was varied between 3% and 8% per year (appendix 4 pp 28–29). Finally, the lifespans of on-premises water and sanitation technologies were shortened from more than ten years to seven for the upper estimates to reflect the climate-related risks of increased droughts and floods that could undermine those assets.^21^

### Benchmark analysis

To gauge financial feasibility, the estimated costs were compared to four relevant expenditure benchmarks: capital expenditure in health by governments and donors, current health expenditure by governments, WASH expenditure by governments, and aid disbursements for WASH. Country-level per-capita estimates from secondary sources^22^, ^23^, ^24^ were used to compute population-adjusted LDC means (appendix 4 pp 30–31).

### Role of the funding source

The funders of the study had no role in study design, data collection, data analysis, data interpretation, or writing of the report.

## Results

Estimated financial costs to achieve full coverage of WASH and waste services in the 46 UN designated LDCs' public health-care facilities are summarised in table 1. The incremental cost beyond current spending levels is $6·5 billion to $9·6 billion from 2021 to 2030. The capital cost is $2·9 billion to $4·8 billion, or a mean of $0·24–0·40 per capita, per year. The recurrent cost over ten years is $3·6 billion to $4·8 billion, increasing from $0·10 per capita in 2021 to $0·39–0·60 (baseline $0·51) per capita in 2030. The undiscounted (fiscal) costs are $9·8 billion to $11·2 billion.

**Table 1:** Incremental cost to reach full water, sanitation, hygiene, and waste service coverage in the least-developed countries' public health-care facilities (2020 US$), 2021–30

	**Total cost (US$ billions)**	**Capital cost (US$ billions)**	**Recurrent cost (US$ billions)**	**Average annual capital cost per capita (US$)**	**Annual recurrent cost per capita in 2021 (US$)**	**Annual recurrent cost per capita in 2030 (US$)**
Baseline	7·9	3·6	4·3	0·30	0·10	0·51
Lower estimate	6·5	2·9	3·6	0·24	0·10	0·39
Upper estimate	9·6	4·8	4·8	0·40	0·10	0·60

The distribution of the baseline estimates over the four services, facility settings, and facility types are shown in table 2. Waste management costs are greatest at $3·7 billion (46·6% of the total), followed by $1·8 billion (23·1%) for sanitation, $1·5 billion (19·5%) for water, and $845 million (10·7%) for hygiene. Waste management's predominance reflects its high per-facility costs (table 3) and low baseline coverage in the LDCs. This service ranking is maintained across most facility settings and types. However, hospitals require considerably more investment in hygiene than in water or sanitation.

**Table 2:** Incremental cost to reach full coverage of water, sanitation, hygiene, and waste services in the least-developed countries' public health-care facilities by service, geography, and facility type (baseline estimates), 2021–30

		**All facilities**		**Urban facilities**		**Rural facilities**		**Hospitals**		**Non-hospitals**		**Number of facilities**	**Share of facilities**
		Cost (US$ billions)	Share of total cost	Cost (US$ billions)	Share of total cost	Cost (US$ billions)	Share of total cost	Cost (US$ billions)	Share of total cost	Cost (US$ billions)	Share of total cost		
Total cost		7·9	100·0%	2·5	32·3%	5·3	67·7%	0·5	6·3%	7·4	93·7%	..	..
Service													
	Water	1·5	19·5%	0·4	4·8%	1·2	14·7%	0·1	0·7%	1·5	18·9%	..	..
	Sanitation	1·8	23·1%	0·5	6·3%	1·3	16·8%	0·1	1·0%	1·7	22·1%	..	..
	Hygiene	0·8	10·7%	0·3	3·5%	0·6	7·3%	0·1	1·5%	0·7	9·2%	..	..
	Waste management	3·7	46·6%	1·4	17·7%	2·3	28·9%	0·2	3·1%	3·4	43·5%	..	..
Geography													
	Urban	2·5	32·3%	..	..	..	..	0·2	3·1%	2·3	29·2%	48 105	33·1%
	Rural	5·3	67·7%	..	..	..	..	0·3	3·2%	5·1	64·5%	97 260	66·9%
Facility type													
	Hospital	0·5	6·3%	0·2	3·1%	0·3	3·2%	..	..	..	..	5583	3·8%
	Non-hospital	7·4	93·7%	2·3	29·2%	5·1	64·5%	..	..	..	..	139 782	96·2%

**Table 3:** Summary of per-facility capital costs and recurrent costs to meet basic water, sanitation, hygiene, and waste service standards in the least-developed countries (2020 US$)

	**Capital costs**	**Recurrent costs**
**Water**		
Non-hospital, rural, piped	5757 (2125–23 750); 38	2000 (500–5289); 35
Non-hospital, rural, on premises	15 601 (6875–28 726); 38	1700 (500–4500); 35
Non-hospital, urban, piped	5000 (2000–9000); 37	1500 (500–3030); 33
Non-hospital, urban, on premises	17 500 (5000–28 330); 33	1425 (500–3450); 30
Hospital, piped	4500 (2000–20 000); 34	2000 (1200–5000); 25
**Sanitation**		
Non-hospital, septic	12 000 (6000–17 376); 40	855 (350–2000); 30
Non-hospital, sewerage	8700 (5000–13 500); 25	300 (150–600); 21
Hospital, septic	18 000 (10 000–30 000); 34	2050 (808–3500); 28
Hospital, sewerage	10 000 (7000–24 000); 25	1000 (600–2006); 20
**Hygiene**		
Non-hospital	1200 (463–3500); 38	330 (200–950); 34
Hospital	2500 (1107–6690); 34	1500 (403–3000); 29
**Waste management**		
Non-hospital	10 159 (3000–15 000); 38	1750 (500–3918); 30
Hospital	21 000 (15 000–50 000); 34	4250 (1500–10 500); 28

Data are median (IQR); n. n is the number of least-developed countries for which cost data were reported on the per-facility cost survey.

**Figure 1:**
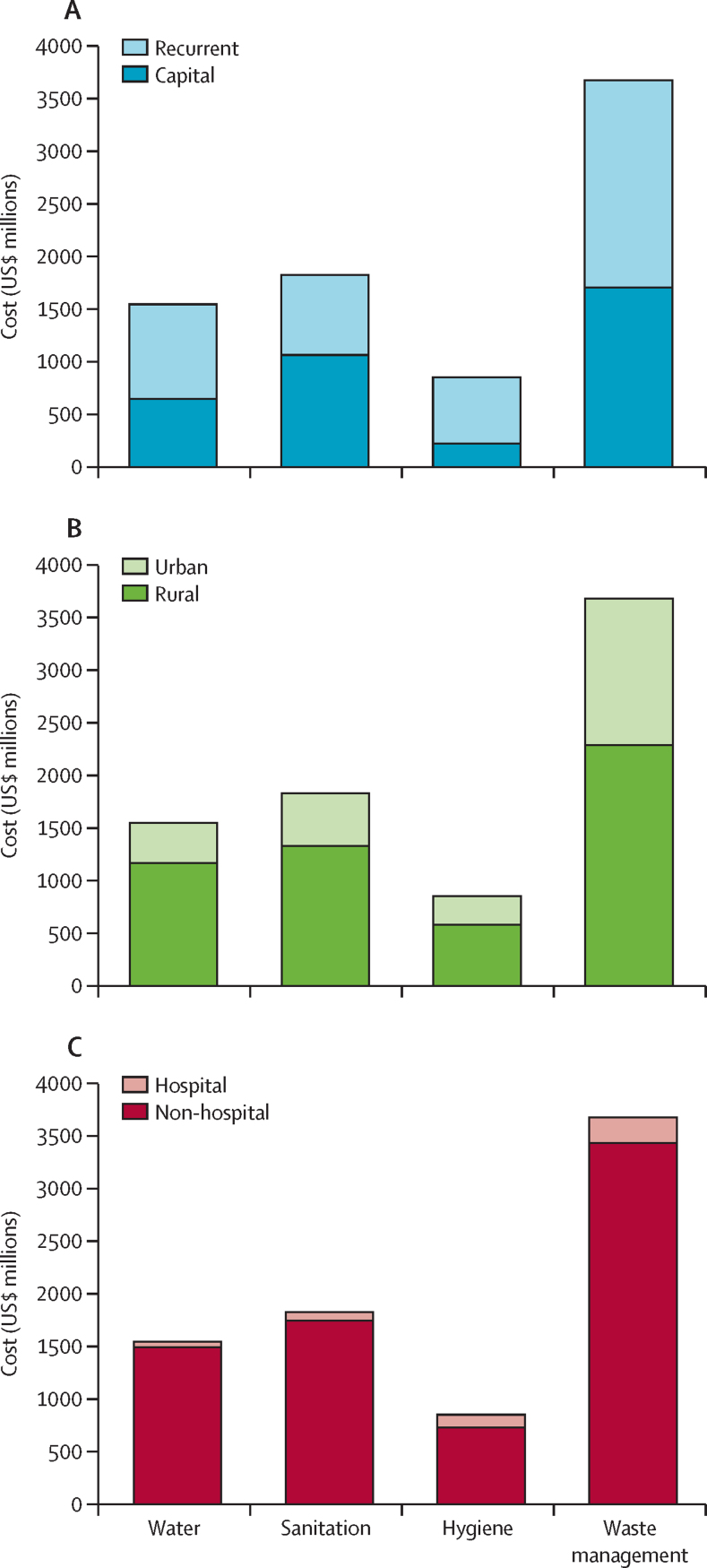
Total costs of meeting basic water, sanitation, hygiene, and waste service levels in the least-developed countries' public health facilities, by service (A) Service costs by capital and recurrent portions. (B) Service costs between rural and urban facilities. (C) Service costs between non-hospital facilities and hospitals.

Sanitation is the most capital-intensive service and the only one for which the majority of costs is for capital investment (figure 1A). For all four services costs are concentrated in rural facilities (figure 1B) and non-hospital facilities (figure 1C). Despite differences in coverage, the distributions of costs across contexts and facility types are driven almost entirely by how facilities were sorted into the four modelled profiles (appendix 4 pp 13–19). 97 260 (67%) of the 145 365 facilities in rural areas account for $5·3 billion (68%) of the $7·9 billion of costs, and the 139 782 (96%) facilities classified as non-hospitals account for $7·4 billion (94%) of costs. Even in urban areas, hospitals only account for $247 million (10%) of $2·5 billion of costs.

**Figure 2:**
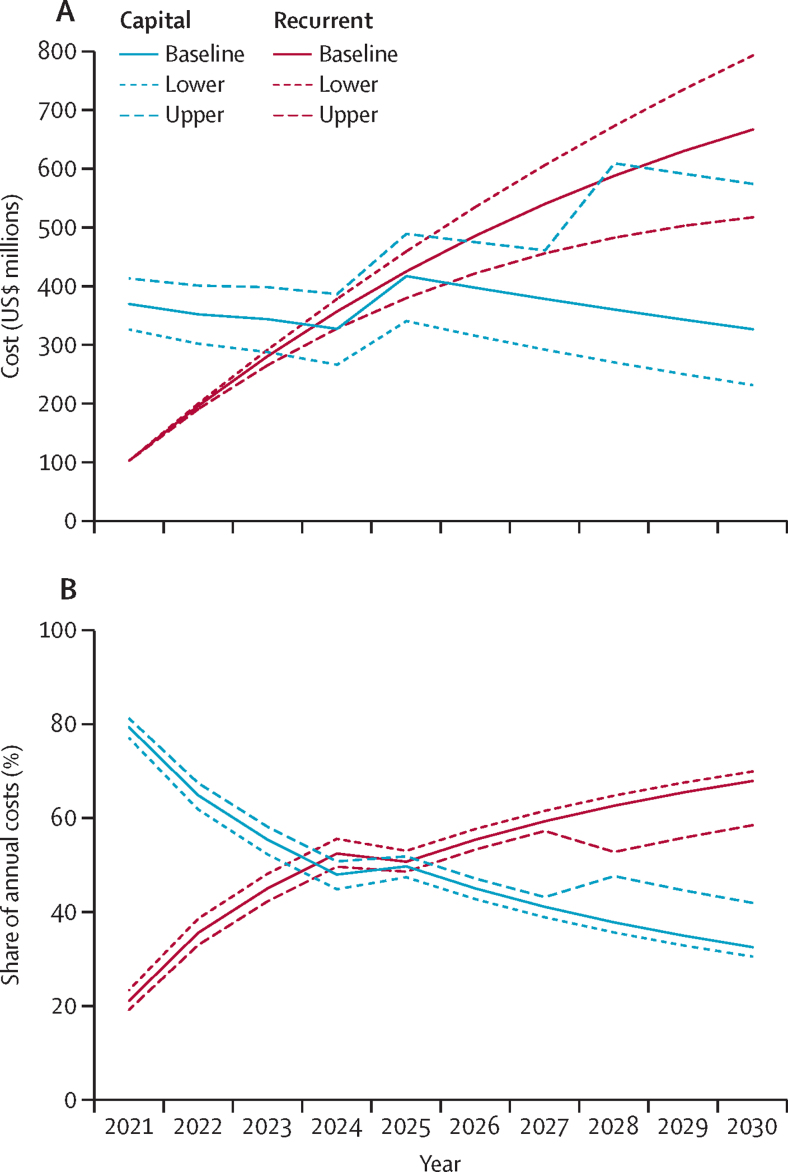
Annual costs (baseline, lower, and upper estimate) of meeting basic water, sanitation, hygiene, and waste service levels in the least-developed countries' public health facilities, 2021–30 (A) Annual capital and recurrent costs. (B) Capital and recurrent shares of annual costs.

Annual recurrent costs grow steadily, from $103 million in 2021 to $516 million to $791 million in 2030, depending on the discount rate applied. Meanwhile, yearly capital costs initially decrease over time and then spike when assets start requiring replacement (figure 2A). As recurrent costs mount, capital's share of annual costs decreases substantially, at times attenuated or reversed by the advent of asset replacement (figure 2B).

Of the parameters varied in the sensitivity analysis (appendix 4 pp 28–29), the discount rate accounted for the greatest deviations from the baseline estimates, followed by the share of per-facility costs assigned to sub-standard facilities and asset lifespan. All three parameters have similar effects on capital costs. On their own, the range of discount rates applied changes total costs by more than 10% in each direction, whereas the other parameters' individual impacts amount to less than 5% of the baseline estimates. Only the discount rate affects recurrent costs. Asset lifespans were varied based on one-tail risks associated with climate change, whose impacts on their own are estimated to increase total costs by nearly $330 million.

The estimated costs are small or moderate compared to expenditure benchmarks (appendix 4 pp 30–31). The mean annual additional capital cost per capita is estimated at $0·30, equal to nearly 20% of the $1·56 per capita invested in 2018 in health capital by 23 LDC governments ($0·80) and their development partners ($0·76). Meanwhile, current health expenditure by 44 LDC governments was $10·17 per capita in 2018 (US$ 2018), meaning limited fiscal space will be needed to cover the estimated additional recurrent costs of $0·10 to $0·51 per capita per year, between 2021 and 2030. Compared with existing expenditure on WASH, the mean annual cost of $0·65 per capita (capital and recurrent) would represent about a one-fifth increase on the $3·09 per capita 22 LDC governments already spend on WASH, or a similar increment on the $3·01 per capita all 46 LDCs received in aggregate as aid for WASH in 2019.^24^

## Discussion

The cost of reaching full coverage of basic WASH and waste management in existing public health-care facilities in LDCs is estimated to be $6·5 billion to $9·6 billion from 2021 to 2030. To our knowledge, this is the first study to quantify the costs of achieving global targets for WASH and waste services in health facilities for a large group of priority countries. Relative to investment needs to reach the health SDGs, which amount to $58 per capita by 2030 (US$ 2014),^14^ the less than $1 per capita required annually to meet basic levels of WASH and waste services in health facilities is minimal. Meeting these basic levels would also require only modest increases to existing health and WASH spending in LDCs.

These findings add to the available evidence on resource needs for achieving global goals for health (SDG 3) and WASH (SDG 6). The resources are needed even if the estimated $193 billion (US$ 2015) required from 2015 to 2030 to achieve the basic WASH service level for households in the LDCs are fully realised (Hutton G, unpublished). The investments needed for households to have basic services would probably not diminish the expected costs for health-care facilities or other institutions because the household-cost estimates did not include any shared costs such as the expansion of piped water or sewerage networks.^12^

This study also helps to unpack health infrastructure's share of the substantial resource requirements for achieving SDG 3, as well as highlights the need to improve existing health infrastructure, which previous analyses minimally address.^14^, ^15^ Given the concentration of additional needs in rural and non-hospital facilities, scaling up investment for WASH and waste services in public health facilities furthers the equity-centred and primary health care-centred post-2015 development agenda for health.^25^

The costs presented here are based on imperfect data sources and thus do not have a high degree of precision. The lower and upper estimates were designed, in part, to account for uncertainty in the underlying coverage data and in the magnitude of investment needs to improve sub-standard facilities that had some existing services. This uncertainty is inherent in the per-facility cost data, which were collected through country consultations and thus were for some countries based on real project costs or on opinions of country experts (or both). Although respondents were instructed to report average costs that accounted for within-country variability, it was not feasible to assess how rigorously they did so. However, the potential bias is partially mitigated by the fixed nature of many of the capital needs (eg, even the smallest facilities require at least two toilets and a reliable, safe source of water to meet basic service-level guidelines) and the fact that facility size might not always correlate with utilisation and, therefore, recurrent costs.

In general, the analysis probably underestimates the global costs for WASH and waste services in public health-care facilities. First, the estimates do not include capital maintenance, which is often included in lifecycle cost analysis for WASH services. Capital maintenance was excluded because the modelling covered a ten-year period rather than the full lifecycles of all assets, and there is minimal evidence on the magnitude and frequency of capital maintenance needs. Second, the scope of the analysis was limited by data availability and, consequently, excludes environmental cleaning and cross-cutting activities such as training, supervision, mentoring, and monitoring and evaluation. Furthermore, costs were only estimated for LDCs due to sparse coverage data for other countries. Although the LDCs have the lowest service coverage, the magnitude of needs elsewhere is probably greater given the large populations and numbers of health facilities in middle-income countries such as China, Brazil, India, Indonesia, and Nigeria. Findings from a 2020 study in India support this hypothesis.^26^ Moreover, only existing facilities were included in the analysis, whereas countries are expected to build many more facilities to achieve SDG 3,^14^ all of which will entail WASH-related and waste-related investments. The costs of improving hygiene behaviours, most notably through the proven multi-modal implementation strategy for hand hygiene,^27^ were also excluded due to the scarcity of data. Additionally, fulfilling the spirit of the World Health Assembly resolution might require exceeding the basic service levels to ensure, for example, the universal safe management of water and sanitation systems and fully meet infection prevention and control and quality of care needs. Future studies that incorporate all WASH and waste services, more countries, and higher service levels will undoubtedly estimate greater total costs. A comprehensive cost estimate for universal access to WASH ought to account for these considerations and resource needs in other institutional settings, such as schools.

Understanding resource needs for WASH and waste services in health facilities is only one step towards implementation. Only 25% of national budgets have line items for WASH and waste management in health facilities,^23^ and there is little published evidence on how it is otherwise financed. To build and sustain services in perpetuity, countries will need to plan and allocate resources within their annual budget cycle, regularly monitor WASH and waste services and spending, and strengthen the enabling environment for the private sector to finance and deliver these services, where appropriate. This analysis assumed that countries will sustain existing services, but in practice spending might not be sufficient to maintain coverage or underlying assets. Because half or more of the costs of increasing coverage will arise from regular operating and maintenance activities, governments, donors, and facilities should collaborate to ensure all new capital investments are accompanied by commitments and processes to ensure funding for recurrent needs. Failure to do so could lead to a flurry of upfront investment followed by rapid service degradation, which would in turn require even greater future investment to replace or rehabilitate neglected assets.

The countries classified as LDCs are diverse. Although some LDCs might be able to increase or reallocate domestic financing to address these needs, those that are affected by conflict, fragility, or limited fiscal capacity will require substantial efforts to prioritise funding for such investments. External funding will remain critical in these contexts, and there are many opportunities to channel humanitarian assistance to more durable health and WASH infrastructure rather than temporary emergency services.

Currently, the lack of basic WASH and waste services in the LDCs causes numerous harms, including hampering an effective response to COVID-19, compromising service quality, and contributing to antimicrobial resistance. These service gaps also undermine fundamental human rights enshrined in various UN and member state documents.^28^ As cross-cutting functions that involve multiple ministries and generate many positive externalities, WASH and waste services in health facilities are often chronically underfunded without explicit prioritisation by governments and partners, as with other common goods for health.^29^, ^30^ Within the health sector, resources need to be prioritised as part of overall investments in universal health coverage and health security-oriented reforms.

There also need to be mechanisms for health officials to coordinate (and even jointly budget) with counterparts in other relevant sectors. For example, the needs and preferences of community members, health-care workers, and educators could collectively inform decisions about where to prioritise new investments in water and sanitation infrastructure and guide technology choices, thereby increasing the likelihood that institutions benefit alongside households from new or improved systems. Prioritisation is also important within health facilities given that some rooms or wards, such as for maternity, can have poorer WASH and waste services, but greater needs and infection risks, than others.^31^ Finally, roles, responsibilities, and lines of accountability for the financing, operations, and maintenance of WASH and waste services in health facilities need to be clearly articulated and commonly understood across levels of government.

Despite their shortcomings, the cost estimates for WASH and waste services in public health-care facilities provide an evidence-based starting point for determining the resources needed to address a harmful health system deficit in the world's poorest countries, as well as indicate that the additional financing needs are modest relative to existing levels of spending on health and WASH. The findings can inform ongoing efforts for smart investments in the COVID-19 response and recovery, as well as encourage greater attention to basic infrastructure in the long run as countries seek to invest in greener and more resilient health systems. To further advance dialogue, governments and their partners should undertake tailored national and local cost analyses to inform routine planning and budgeting, as well as systematise practices for sound asset management.

For more on **household data** see washdata.org/data/household

For more on **health-care facilities coverage data** see washdata.org/data/healthcare

## Data sharing

Due to confidentiality agreements with respondents to UNICEF's survey, the per-facility capital and recurrent costs data cannot be made publicly available. A description of the database and survey are provided in appendix 4 (pp 5–12), and those seeking additional information or access should contact Jorge Alvarez-Sala Torreano (jalvarezsala@unicef.org). UNICEF will evaluate any requests for access on a case-by-case basis. All other data used in this study were from publicly available sources or are catalogued in appendix 4 (pp 19, 21–22, 25–28).

## Declaration of interests

MC reports personal fees from WHO during the conduct of the study and from Results for Development, ThinkWell, and the World Bank outside the submitted work. SM reports personal fees from WHO during the conduct of the study and from Evidence Action and Vysnova Partners outside the submitted work. ONH reports personal fees from WHO during the conduct of the study. RJ reports grants from Agence Française de Développement, the Bill & Melinda Gates Foundation, Government of the Netherlands Ministry of Foreign Affairs, UN-Water Inter-Agency Trust Fund, United Kingdom Foreign, Commonwealth & Development Office, and Swiss Agency for Development and Cooperation, both during the conduct of the study and outside the submitted work. All other authors declare no competing interests.

## Supplementary Materials

Arabic translation of the abstract

French translation of the abstract

Portuguese translation of the abstract

## Footnotes

† Environmental cleaning was excluded from the cost analysis due to insufficient data on existing levels of coverage.
