# Supplementary material for: Estimating the cost of achieving basic water, sanitation, hygiene, and waste management services in public health-care facilities in the 46 UN designated least-developed countries: a modelling study
Source: Lancet Glob Health. 2022 Apr 6;10(6):e840–9. doi: 10.1016/S2214-109X(22)00099-7 (PMC9090898; doi:10.1016/S2214-109X(22)00099-7)
Supplement: Arabic translation of the abstract [file mmc1.pdf]

THE LANCET
Global Health

Supplementary appendix 1

This translation in Arabic was submitted by the authors and we reproduce it as supplied. It has not been peer reviewed. *The Lancet*'s editorial processes have only been applied to the original in English, which should serve as reference for this manuscript.

تم تقديم هذه الترجمة باللغة العربية من قبل المؤلفين ونعيد إنتاجها كما هو مُقدم. إنها لم تخضع لاستعراض الأقران. تم تطبيق عمليات تحرير لانسيت فقط على النص الأصلي باللغة الإنجليزية، والذي يجب أن يكون بمثابة مرجع لهذه المخطوطة.

Supplement to: Chaitkin M, McCormick S, Alvarez-Sala Torreano J, et al. Estimating the cost of achieving basic water, sanitation, hygiene, and waste management services in public health-care facilities in the 46 UN designated least-developed countries: a modelling study. *Lancet Glob Health* 2022; published online April 6. https://doi.org/10.1016/S2214-109X(22)00099-7.

دراسة لتحديد نماذج تقدير تكلفة توفير المياه وخدمات الصرف الصحي والنظافة الصحية الأساسية وإدارة النفايات في منشآت الرعاية الصحية العامة في البلدان الستة والأربعين التي تعتبرها الأمم المتحدة ضمن أقل البلدان نموا

موجز

خلفية عامة: يعاني عدد هائل من منشآت الرعاية الصحية العامة في البلدان منخفضة ومتوسطة الدخل من نقص في المياه وخدمات الصرف الصحي والنظافة الصحية وإدارة النفايات. وتضع هذه الدراسة تقديرات لتكاليف توفير تلك الخدمات للجميع في منشآت الصحة العامة الحالية في البلدان الستة والأربعين التي تعتبرها الأمم المتحدة ضمن أقل البلدان نموا.

النُهُج المستخدمة: في هذه الدراسة، تم تحديد عدد المنشآت التي تعاني من نقص المياه وخدمات الصرف الصحي والنظافة الصحية بالجمع بين العدد المُعلن للمنشآت العامة من جهة، وبين تقديرات توفير تلك الخدمات الأساسية من جهة أخرى. وتم جمع بيانات التكاليف الاستثمارية والتكاليف الجارية اللازمة لتقديم الخدمات الأساسية، حسب المنشآت، من خلال مسح استقصائي شارك فيه خبراء ومسؤولو المياه والصرف الصحي والنظافة الصحية في تلك البلدان خلال الفترة بين 24 سبتمبر/أيلول و 24 ديسمبر/كانون الأول2020 . ووضعت نماذج لتقديرات التكلفة الأساسية واُحتسبت نسبة خصم قدرها 5% سنويا، وجرى تعديل الافتراضات الرئيسية لوضع حدود دنيا وعليا للتقديرات، بما في ذلك تعديل نسبة الخصم إلى 8% و 3% سنويا على التوالي.

الاستنتاجات: خلصت الدراسة إلى أن هناك حاجة إلى ما يقدر بنحو 6.5 – 9.6 مليارات دولار خلال الفترة من عام 2021 وحتى عام 2030 لتحقيق التغطية الكاملة للمياه وخدمات الصرف الصحي والنظافة الصحية العامة الأساسية في منشآت الصحة العامة في أقل البلدان نموا. وتتراوح التكاليف الرأسمالية من 2.9 مليار دولار إلى 4.8 مليارات دولار، والتكاليف المتكررة من 3.6 إلى 4.8 مليارات دولار خلال هذه الفترة الزمنية. وفي المتوسط، هناك حاجة إلى 0.24 دولار –0.40 دولار للفرد من الاستثمارات الرأسمالية كل عام، في حين من المتوقع أن ترتفع تكاليف الصيانة والتشغيل السنوية من 0.10 دولار في 2021 إلى 0.39 –0.60 دولار في 2030.وتمثل إدارة النفايات أكبر حصة من التكاليف، حيث تتطلب 3.7 مليارات دولار (46.6% من الإجمالي) في التقديرات الأساسية، يليها الصرف الصحي بتكلفة قدرها 1.8 مليار دولار (23.1%)، والمياه بتكلفة قدرها 1.5 مليار دولار (19.5%)، وأخيراً النظافة الصحية بتكلفة قدرها 845 مليون دولار (10.7%). وتكون الاحتياجات أكبر من ذلك في المنشآت الأخرى غير المستشفيات (7.4 مليارات دولار [94%] من 7.9 مليارات دولار) والمنشآت الواقعة في المناطق الريفية (5.3 مليارات دولار [68%]).

التفسير: سيلزم زيادة الاستثمارات لتوفير المياه وخدمات الصرف الصحي والنظافة الصحية الأساسية وإدارة النفايات للجميع في منشآت الصحة العامة. وتُعد الاحتياجات المالية متواضعة مقارنة بحجم الإنفاق العام الحالي على الصحة والمياه والصرف الصحي والنظافة الصحية، ومن شأن تحسين تغطية الخدمات أن يحقق منافع صحية كبيرة. وللحفاظ على تلك الخدمات ومنع تدهور حالة المنشآت واستبدالها في وقت مبكر، سيتعين على البلدان أن ترصد في موازناتها بانتظام موارد لتغطية تكاليف تشغيل وصيانة معدات ومنشآت المياه وخدمات الصرف الصحي والنظافة الصحية للجميع وإدارة النفايات.

التمويل: منظمة الصحة العالمية (بما في ذلك المنح الأساسية من حكومات اليابان وهولندا والمملكة المتحدة)، والبنك الدولي (بما في ذلك منحة أساسية من الشراكة العالمية للأمن المائي وخدمات الصرف الصحي)، واليونيسف.

حقوق الملكية: © منظمة الصحة العالمية 2022؛ المرخص له (أوفيير). هذه مقالة مفتوحة المصدر وهي تُنشر بموجب رخصة المشاع الإبداعي (CC BY 3.0 IGO) التي تسمح باستخدام وتوزيع واستنساخ غير مقيد في أي وسيلة، شريطة ذكر العمل الأصلي بشكل صحيح. وعند استخدام ما ورد في هذه المقالة، لا ينبغي أن يكون هناك أي إيحاء بأن منظمة الصحة العالمية تؤيد أي منظمة أو منتجات أو خدمات محددة، ولا يُسمح باستخدام شعارها. ويجب الحفاظ على هذا الإشعار جنبا إلى جنب مع عنوان URL الأصلي الخاص بالمقالة.
