# Supplementary material for: Estimating the cost of achieving basic water, sanitation, hygiene, and waste management services in public health-care facilities in the 46 UN designated least-developed countries: a modelling study
Source: Lancet Glob Health. 2022 Apr 6;10(6):e840–9. doi: 10.1016/S2214-109X(22)00099-7 (PMC9090898; doi:10.1016/S2214-109X(22)00099-7)
Supplement: French translation of the abstract [file mmc2.pdf]

THE LANCET
Global Health

Supplementary appendix 2

This translation in French was submitted by the authors and we reproduce it as supplied. It has not been peer reviewed. *The Lancet*'s editorial processes have only been applied to the original in English, which should serve as reference for this manuscript.

Cette traduction en français a été proposée par les auteurs et nous l'avons reproduite telle quelle. Elle n'a pas été examinée par des pairs. Les processus éditoriaux du *Lancet* n'ont été appliqués qu'à l'original en anglais et c'est cette version qui doit servir de référence pour ce manuscrit.

Supplement to: Chaitkin M, McCormick S, Alvarez-Sala Torreano J, et al. Estimating the cost of achieving basic water, sanitation, hygiene, and waste management services in public health-care facilities in the 46 UN designated least-developed countries: a modelling study. *Lancet Glob Health* 2022; published online April 6. https://doi.org/10.1016/S2214-109X(22)00099-7.

Estimation du coût de prestation des services d’eau, d’assainissement et d’hygiène et de gestion des déchets de base dans les établissements de santé publique des 46 pays désignés « les moins avancés » par l’ONU : étude de modélisation

Résumé

Contexte général : Un nombre alarmant d’établissements de santé publique des pays à revenu faible ou intermédiaire sont dépourvus de services d’eau, d’assainissement et d’hygiène (WASH) et de gestion des déchets de base. La présente étude évalue ce qu’il en coûterait pour assurer la prestation de l’ensemble de ces services dans les établissements existants de santé publique des 46 pays désignés « les moins avancés » (PMA) par l’ONU.

Méthodes : Dans cette étude de modélisation, les établissements présentant des lacunes en ces matières ont été recensés en combinant les données publiées dénombrant les établissements publics et les estimations de la couverture des services WASH et de gestion des déchets de base. Les données sur les dépenses d’équipement et de fonctionnement requises par établissement et par pays pour la prestation de ces services de base ont été recueillies dans le cadre d’un sondage réalisé auprès des experts et des représentants officiels des services WASH entre le 24 septembre et le 24 décembre 2020. Les estimations des coûts de base ont été modélisées et actualisées de 5 % par an. Les principales hypothèses ont été ajustées pour produire des estimations inférieure et supérieure, en ajustant le taux d’actualisation à 8 % et à 3 % par an respectivement.

Résultats : On a estimé qu’il faudrait entre 6,5 et 9,6 milliards de dollars de 2021 à 2030 pour atteindre la couverture totale des besoins en matière de services WASH de base dans les établissements de santé publique des PMA. Les dépenses d’équipement oscillent entre 2,9 et 4,8 milliards, et les dépenses de fonctionnement oscillent entre 3,6 et 4,8 milliards sur cette période. Il faudrait investir en moyenne 0,24 à 0,40 dollar par habitant chaque année, alors que les coûts d’entretien et de fonctionnement devraient passer de 0,10 dollar par habitant en 2021 à 0,39–0,60 dollar par habitant en 2030. La gestion des déchets représente la plus grande part des coûts, s’établissant à 3,7 milliards (46,6 % du total) dans les estimations de référence, suivie par l’assainissement (1,8 milliard, ou 23,1 %), l’approvisionnement en eau (1,5 milliard, ou 19,5 %) et l’hygiène (845 millions, ou 10,7 %). Les besoins sont plus importants dans les établissements non hospitaliers (7,4 milliards, ou 94 % du total de 7,9 milliards) et dans les établissements de zones rurales (5,3 milliards, ou 68 %).

Interprétation : Les investissements devront être augmentés pour assurer la pleine couverture des besoins de base en matière de services WASH et de gestion des déchets dans les établissements de santé publique. Les besoins financiers sont modestes comparativement aux dépenses globales actuelles consacrées à la santé et aux services WASH, et une couverture améliorée procurera des avantages sensibles en matière de santé. Pour assurer la pérennité des services et éviter la dégradation et les remplacements précoces, les pays devront prévoir des ressources budgétaires régulières pour le fonctionnement et l’entretien des équipements de services WASH et de gestion des déchets.

Financement : OMS (y compris une aide financière sous-jacente du Japon, des Pays-Bas et du Royaume-Uni), Banque mondiale (y compris une aide financière sous-jacente du Partenariat mondial pour la sécurité de l’eau et l’assainissement) et UNICEF.

Copyright : © 2022 Organisation mondiale de la Santé ; titulaire des droits : Elsevier. L’utilisation de ce document en accès libre est soumise aux conditions de la licence CC BY 3.0 IGO qui en autorise l’utilisation, la diffusion et la reproduction sous toute forme, à condition qu’il soit cité de manière appropriée. Quelle que soit l’utilisation qui en sera faite, il ne devra pas être suggéré que l’OMS approuve une organisation, des produits ou des services particuliers. L’utilisation du logo de l’OMS est interdite. Le présent avis devrait être conservé avec l’adresse URL originale de l’article.
