# Supplementary material for: Estimating the cost of achieving basic water, sanitation, hygiene, and waste management services in public health-care facilities in the 46 UN designated least-developed countries: a modelling study
Source: Lancet Glob Health. 2022 Apr 6;10(6):e840–9. doi: 10.1016/S2214-109X(22)00099-7 (PMC9090898; doi:10.1016/S2214-109X(22)00099-7)
Supplement: Portuguese translation of the abstract [file mmc3.pdf]

THE LANCET
Global Health

Supplementary appendix 3

This translation in Portuguese was submitted by the authors and we reproduce it as supplied. It has not been peer reviewed. The Lancet's editorial processes have only been applied to the original in English, which should serve as reference for this manuscript.

Esta tradução em português foi submetida pelos autores e nós não fizemos quaisquer alterações. Esta versão não foi revista por pares. O processo editorial do The Lancet só foi aplicado à versão original em inglês, que deve servir como referência para este artigo.

Supplement to: Chaitkin M, McCormick S, Alvarez-Sala Torreano J, et al. Estimating the cost of achieving basic water, sanitation, hygiene, and waste management services in public health-care facilities in the 46 UN designated least-developed countries: a modelling study. *Lancet Glob Health* 2022; published online April 6. https://doi.org/10.1016/S2214-109X(22)00099-7.

Estimativa do custo de conseguir ter serviços básicos de água, saneamento, higiene e gestão de resíduos nas instalações de saúde pública nos 46 países designados pela ONU como menos desenvolvidos: um estudo de modelação

Resumo

Antecedentes: Um número alarmante de instalações de saúde pública em países com rendimentos baixos e médios carece de serviços básicos de água, saneamento, higiene (WASH) e de gestão de resíduos. Este estudo estima os custos de conseguir uma cobertura total dos serviços básicos de WASH e de gestão de resíduos nas instalações de saúde pública existentes nos 46 países designados pela ONU como menos desenvolvidos (PMD).

Métodos: Neste estudo de modelação, as instalações necessitadas foram quantificadas através da combinação de contagens publicadas de instalações públicas com a cobertura básica estimada de WASH e serviços de gestão de resíduos. O capital necessário por instalação específico para cada país e os custos recorrentes para fornecer serviços básicos foram recolhidos através de um inquérito feito aos peritos e funcionários dos serviços de WASH do país entre 24 de setembro e 24 de dezembro de 2020. As estimativas dos custos de base foram modeladas e descontadas a 5% ao ano. Os principais pressupostos foram ajustados para produzir estimativas inferiores e superiores, incluindo o ajuste da taxa de desconto para 8% e 3% ao ano, respetivamente.

Constatações: Estima-se que são necessários US$6,5 mil milhões a US$9,6 mil milhões de 2021 a 2030 para conseguir uma cobertura total dos serviços básicos de WASH nas instalações de saúde pública nos países menos desenvolvidos. Os custos de capital são de US$2,9 mil milhões a US$4,8 mil milhões e os custos recorrentes são de US$3,6 mil milhões a US$4,8 mil milhões durante esse período de tempo. Em média, são necessários US$0,24 a US$0,40 per-capita em investimentos de capital a cada ano, enquanto os custos anuais de manutenção e operação devem aumentar de US$0,10 em 2021 para US$0,39 a US$0,60 em 2030. A gestão de resíduos é responsável pela maior parte dos custos, necessitando de US$3,7 mil milhões (46,6% do total) nas estimativas de base, seguindo-se o saneamento com US$1,8 mil milhões (23,1%), a água com US$1,5 mil milhões (19,5%), e a higiene com US$845 milhões (10,7%). As necessidades são maiores para instalações não-hospitalares (US$7,4 mil milhões [94%] de um total de US$7,9 mil milhões) e para instalações em áreas rurais (US$5,3 mil milhões [68%]).

Interpretação: O investimento terá de aumentar para alcançar uma cobertura total dos serviços básicos de WASH e de gestão de resíduos nas instalações de saúde pública. As necessidades financeiras são modestas em comparação com as despesas atuais globais em saúde e WASH, e uma melhor cobertura dos serviços irá produzir benefícios de saúde significativos. Para manter os serviços e evitar a sua degradação e necessidade de substituição precoce, os países terão que orçamentar rotineiramente as operações e a manutenção dos serviços de WASH e dos ativos de gestão de resíduos.

Financiamento: OMS (incluindo subsídios subjacentes dos governos do Japão, Países Baixos e Reino Unido), Banco Mundial (incluindo um subsídio subjacente da Parceria Global para a Segurança da Água e Saneamento), e UNICEF.

Copyright: © 2022 Organização Mundial de Saúde; licenciado Elsevier. Este é um artigo de acesso aberto distribuído nos termos da licença CC BY 3.0 IGO que permite a utilização, distribuição e reprodução sem restrições em qualquer meio, desde que o trabalho original seja devidamente citado. Em qualquer utilização deste artigo, não deve haver nenhuma sugestão de que a OMS endossa qualquer organização, produtos ou serviços específicos. A utilização do logotipo da OMS não é permitida. Este aviso deve ser preservado juntamente com o URL original do artigo.
