# Supplementary material for: Estimating the cost of achieving basic water, sanitation, hygiene, and waste management services in public health-care facilities in the 46 UN designated least-developed countries: a modelling study
Source: Lancet Glob Health. 2022 Apr 6;10(6):e840–9. doi: 10.1016/S2214-109X(22)00099-7 (PMC9090898; doi:10.1016/S2214-109X(22)00099-7)
Supplement: Supplementary appendix 4 [file mmc4.pdf]

THE LANCET
Global Health

Supplementary appendix 4

This appendix formed part of the original submission and has been peer reviewed. We post it as supplied by the authors.

Supplement to: Chaitkin M, McCormick S, Alvarez-Sala Torreano J, et al. Estimating the cost of achieving basic water, sanitation, hygiene, and waste management services in public health-care facilities in the 46 UN designated least-developed countries: a modelling study. *Lancet Glob Health* 2022; published online April 6. https://doi.org/10.1016/S2214-109X(22)00099-7.

Supplementary Material

This document expands on the methods and findings described in the main paper.[a] It is organized as follows:

Section 1: Previous cost estimate published by WHO and UNICEF
Section 2: Countries included in the analysis
Section 3: Per-facility costs
Section 4: Number of facilities
Section 5: Quantifying needs
Section 6: Water and sanitation service assumptions
Section 7: Modelling scale-up and asset replacement
Section 8: Sensitivity analysis
Section 9: Benchmark analysis

[a] Readers seeking additional detail to what is provided in this document should please contact the corresponding author (michael@chaitkin.com).

Abbreviations

CAPEX	Capital expenditure
CHAM	Christian Health Association of Malawi
CHOICE	Choosing Methods that are Cost-Effective
CRS	Creditor Reporting System
GGHE-D	general government health expenditure from domestic sources
GHED	Global Health Expenditure Database
GLAAS	Global Analysis and Assessment of Sanitation and Drinking Water
HCF	health care facility
HeRAMS	Health Resources Availability Monitoring System
JMP	WHO/UNICEF Joint Monitoring Programme for Water Supply, Sanitation, and Hygiene
LDC	Least Developed Country
O&M	operations and maintenance
ODA	Official Development Assistance
OECD	Organisation for Economic Co-operation and Development
OWNP	One WASH National Programme (Ethiopia)
SDGs	Sustainable Development Goals
UN	United Nations
UNICEF	United Nations Children's Fund
US$	United States dollars
USAID	United States Agency for International Development
WASH	water, sanitation, and hygiene
WHO	World Health Organization

Section 1: Previous cost estimate published by WHO and UNICEF

This study updates and substantiates a preliminary estimate of US$3·6 billion that WHO and UNICEF published in late 2020 within a broader global progress report on water, sanitation, and hygiene (WASH) and waste management in health care facilities.[1] Based on this study, WHO and UNICEF will issue an update to their global report such that it will no longer contain the previous findings.

The cost survey (see section 3) and preliminary analysis was undertaken under the technical guidance of a steering group co-chaired by UNICEF and WHO, with participation from the World Bank, Water 2020, and WaterAid. Between September and December 2020, a series of virtual steering group meetings were held to discuss and refine the study aims, methodology, and model parameters and assumptions, as well as to jointly review findings and their interpretation. At times, steering group members involved additional experts from within their organizations and beyond to provide inputs, either by joining a steering group meeting or over email. After review and validation with the steering group, the preliminary estimates were presented during a global webinar on December 14, 2020, which was organized as part of the broader dissemination activities for the WHO-UNICEF global progress report.

The estimated costs presented in this study reflect three major methodological updates. First, the earlier estimates included costs for Vanuatu, which graduated from Least Developed Country (LDC) classification in December 2020 and was thereafter removed from the analysis. Second, the earlier estimates relied on a pre-existing dataset for country-level health facility counts that was several years out of date and did not contain data on low-level facilities like clinics and health posts. For this study a more up-to-date and comprehensive dataset was compiled from primary and secondary sources (see section 4). Finally, the earlier estimates included costs for environmental cleaning and other activities, which were ultimately excluded from this study due to concerns about data availability and over-reliance on assumptions in the modelling.

Section 2: Countries included in the analysis

This study includes the 46 countries that the United Nations designated as LDCs as of December 2020 (table S1). According to UN Department of Economic and Social Affairs, LDCs face "severe structural impediments to sustainable development" and "are highly vulnerable to economic and environmental shocks and have low levels of human assets."[2] Inclusion of countries is based on measures of per capita income, human assets (combining health and education indices), and economic and environmental vulnerability.

Table S1. List of countries included in the analysis

Country	UNICEF Operational Region	Population (2020)[3]	Urbanization (2020)[4]
Afghanistan	South Asia	38,928,341	26·0%
Angola	Eastern and Southern Africa	32,866,268	66·8%
Bangladesh	South Asia	164,689,383	38·2%
Benin	West and Central Africa	12,123,198	48·4%
Bhutan	South Asia	771,612	42·3%
Burkina Faso	West and Central Africa	20,903,278	30·6%
Burundi	Eastern and Southern Africa	11,890,781	13·7%
Cambodia	East Asia and Pacific	16,718,971	24·2%
Central African Republic	West and Central Africa	4,829,764	42·2%
Chad	West and Central Africa	16,425,859	23·5%
Comoros	Eastern and Southern Africa	869,595	29·4%
Democratic Republic of the Congo	West and Central Africa	89,561,404	45·6%
Djibouti	Middle East and North Africa	988,002	78·1%
Eritrea	Eastern and Southern Africa	3,546,427	41·3%
Ethiopia	Eastern and Southern Africa	114,963,583	21·7%
The Gambia (Republic of)	West and Central Africa	2,416,664	62·6%
Guinea	West and Central Africa	13,132,792	36·9%
Guinea Bissau	West and Central Africa	1,967,998	44·2%
Haiti	Latin America and the Caribbean	11,402,533	57·1%
Kiribati	East Asia and Pacific	119,446	55·6%
Lao People's Democratic Republic	East Asia and Pacific	7,275,556	36·3%
Lesotho	Eastern and Southern Africa	2,142,252	29·0%
Liberia	West and Central Africa	5,057,677	52·1%
Madagascar	Eastern and Southern Africa	27,691,019	38·5%
Malawi	Eastern and Southern Africa	19,129,955	17·4%
Mali	West and Central Africa	20,250,834	43·9%
Mauritania	West and Central Africa	4,649,660	55·3%
Mozambique	Eastern and Southern Africa	31,255,435	37·1%
Myanmar	East Asia and Pacific	54,409,794	31·1%
Nepal	South Asia	29,136,808	20·6%
Niger	West and Central Africa	24,206,636	16·6%
Rwanda	Eastern and Southern Africa	12,952,209	17·4%
São Tomé and Príncipe	West and Central Africa	219,161	74·4%
Senegal	West and Central Africa	16,743,930	48·1%
Sierra Leone	West and Central Africa	7,976,985	42·9%
Solomon Islands	East Asia and Pacific	686,878	24·7%
Somalia	Eastern and Southern Africa	15,893,219	46·1%
South Sudan	Eastern and Southern Africa	11,193,729	20·2%
Sudan	Middle East and North Africa	43,849,269	35·3%
United Republic of Tanzania	Eastern and Southern Africa	59,734,213	35·2%
Timor-Leste	East Asia and Pacific	1,318,442	31·3%
Togo	West and Central Africa	8,278,737	42·8%
Tuvalu	East Asia and Pacific	11,792	64·0%
Uganda	Eastern and Southern Africa	45,741,000	25·0%
Yemen	Middle East and North Africa	29,825,968	37·9%
Zambia	Eastern and Southern Africa	18,383,956	44·6%

Section 3: Per-facility costs

Country survey

This sub-section describes the origins and nature of the cost data collected by UNICEF between September 24, 2020 and December 24, 2020. In many cases, data were provided on the condition that they would only be used to generate multi-country estimates in a global study—there is no official approval from relevant authorities to publish country-identified cost data. For this reason, no country-specific costs or resource needs estimates are presented in the main paper or in this appendix. Readers seeking additional information about these data should contact Jorge Alvarez-Sala Torreano (jalvarezsala@unicef.org).

A data collection instrument was circulated to the UNICEF Chief of WASH in 59 countries, including all 46 LDCs. Of these, information was received back from 44 countries, including 40 LDCs (table S2). It was originally hoped that the cost analysis would cover all surveyed countries, but due to data limitations (e.g., country-level estimates of WASH and waste service coverage), only the LDCs were ultimately included. Consequently, only data received from LDCs were used in this study. At the time of data collection, there were 47 LDCs; following Vanuatu's graduation from LDC classification in December 2020, it was removed from the cost model, leaving 46 countries. UNICEF is exploring ways to make use of cost data received from non-LDCs to extend the analysis presented here.

Table S2. List of countries surveyed by UNICEF for WASH and waste services cost information

Country	LDC	Response		Country	LDC	Response		Country	LDC	Response
Afghanistan	Yes	Yes		Angola	Yes	Yes		Burundi	Yes	Yes
Benin	Yes	Yes		Burkina Faso	Yes	Yes		Bangladesh	Yes	Yes
Brazil	No	No		Bhutan	Yes	No		Central African Republic	Yes	Yes
China	No	No		Democratic Republic of the Congo	Yes	Yes		Comoros	Yes	Yes
Djibouti	Yes	Yes		Eritrea	Yes	Yes		Ethiopia	Yes	Yes
Guinea	Yes	Yes		The Gambia (Republic of)	Yes	Yes		Guinea-Bissau	Yes	Yes
Haiti	Yes	Yes		Indonesia	No	No		India	No	Yes
Islamic Republic of Iran	No	No		Cambodia	Yes	Yes		Kiribati	Yes	No
Lao People's Democratic Republic	Yes	Yes		Liberia	Yes	Yes		Lesotho	Yes	Yes
Madagascar	Yes	Yes		Mexico	No	No		Mali	Yes	Yes
Myanmar	Yes	Yes		Mozambique	Yes	Yes		Mauritania	Yes	Yes
Malawi	Yes	Yes		Niger	Yes	No		Nigeria	No	Yes
Nepal	Yes	Yes		Pakistan	No	Yes		Philippines	No	No
Papua New Guinea	No	Yes		Rwanda	Yes	Yes		Sudan	Yes	Yes
Senegal	Yes	No		Solomon Islands	Yes	Yes		Sierra Leone	Yes	Yes
Somalia	Yes	Yes		South Sudan	Yes	No		São Tomé and Príncipe	Yes	Yes
Chad	Yes	Yes		Togo	Yes	Yes		Thailand	No	No
Timor-Leste	Yes	Yes		Tuvalu	Yes	No		United Republic of Tanzania	Yes	Yes
Uganda	Yes	Yes		Viet Nam	No	No		Vanuatu	No*	No
Yemen	Yes	Yes		Zambia	Yes	Yes				

*Vanuatu was classified as an LDC at the time of data collection and then graduated in December 2020. The preliminary resource needs estimate within the 2020 global progress report on WASH in health care facilities[1] included Vanuatu, whereas the country is excluded from the findings presented in this study.

The data collection instrument was designed to rapidly capture the main cost drivers for WASH and waste services in health care facilities (table S3), informed by consultations with experts at UNICEF and WHO, and from practical experience supporting the national planning and budgeting exercise for Ethiopia's One WASH National Programme (OWNP) Phase II.[b] Respondents were asked to submit per-facility investments required to achieve *basic* service levels in hospitals and non-hospitals, differentiating between one-time capital costs and annual recurrent costs.[c] All cost data used in this study were provided by survey respondents in 2020 United States dollars.

Table S3. Costed WASH and waste services included in the UNICEF country survey

Service	Facility type	Sub-service category
Water	Non-hospital, rural	Connection to piped source†
Water	Non-hospital, rural	On-premises source
Water	Non-hospital, urban	Connection to piped source†
Water	Non-hospital, urban	On-premises source
Water	Hospital	Connection to piped source†
Sanitation	Non-hospital	Connection to sewerage†
Sanitation	Non-hospital	Septic system
Sanitation	Hospital	Connection to sewerage†
Sanitation	Hospital	Septic system
Hygiene	Non-hospital	NS
Hygiene	Hospital	NS
Waste Management	Non-hospital	NS
Waste Management	Hospital	NS
Cleaning	Non-hospital	NS
Cleaning	Hospital	NS
Other*	Non-hospital	NS
Other*	Hospital	NS

NS = not specified.

*Other costs included one-time and annual costs for WASH-related training, planning, and monitoring activities. These were not incorporated into the analysis.
†Costs for connections to networks only included connection costs from the facility to the network and within the facility, not any costs associated with construction, expansion, operation, or maintenance of the network itself.

Detailed instructions were transmitted to respondents with the data collection instrument. Respondents were explicitly directed to report average costs, mindful of the considerable variability likely to exist in their countries (see Box S1). Beyond the simplified facility types (hospitals, non-hospitals) and locations (urban, rural), no specific assumptions or guidance were provided regarding facility size. It was assumed the respondents accounted for variation in facility sizes when providing average costs.

The instructions recommended that respondents submit the average costs of new infrastructure rather than rehabilitating existing infrastructure unless most facilities in the country only needed rehabilitation. Information in respondent comments accompanying their submissions and subsequent communications with some respondents made it clear that it was too challenging to quickly estimate average costs reflecting different levels of investment needs within a country's facility stock. Instead, respondents consistently provided costs corresponding to new infrastructure, as if facilities had no WASH and waste services. Consequently, additional rules were developed to assign different costs to facilities with "limited" versus "no" services at baseline (see section 5).

Monitoring definitions from the WHO/UNICEF Joint Monitoring Programme for Water Supply, Sanitation, and Hygiene (JMP) for basic service levels for WASH in health care facilities were also provided to respondents, excerpted directly from the original source[5] (figures S1–S5). It was not feasible to assess how rigorously respondents determined average costs. For example, if respondents based non-hospital costs primarily on larger health centres, this study may have overestimated costs associated with smaller clinics and health posts, which were

[b] See https://www.unicef.org/ethiopia/reports/one-wash-national-programme.

[c] The definition of capital and recurrent costs here aligns with the terms "capital expenditure (CapEx)" and "operations and maintenance (O&M)" commonly used in WASH costing studies.

nearly 60% of the non-hospital facilities included in the analysis. Factors mitigating the potential bias are noted in the main paper.

Box S1. Instructions accompanying the data collection instrument

Important notes. Please read carefully before completing the form.

Thank you for being part of this important initiative that aims at making a business case for WASH investments in HCF. This form has been designed to collect key information that will be used to estimate national and global costs for achieving universal access to WASH in HCF. We are aware that some information might not be easily available and that there might be significant variables and variability across the country. What we aim is to collect the average costs based on the available data and your experience in the country.

When estimating the costs, you need to estimate them based on the amounts NEEDED to achieve JMP's basic service level, NOT the average CURRENT investment in HCF. This is very important for countries where the current levels of investments are insufficient to meet the JMP standards of basic service. You might however have some HCFs which meet the national and JMP standards of basic service, and which can be used as a benchmark for establishing the costs.

JMP service level indicators have been included in a separate sheet "JMP reference basic service" for your reference. Further information is available here: https://washdata.org/sites/default/files/documents/reports/2019-04/JMP-2018-core-questions-for-monitoring-WinHCF.pdf.

For CAPEX estimates please consider the costs of moving from JMP's "limited" or "no service" to" basic" service level. Those investments might be different if HCFs require a brand new infrastructure or a mayor rehabilitation of the existing infrastructures. Unless the majority of those limited/no-service HCFs have existing infrastructures that just require rehabilitation, we recommend that you estimate the cost of construction of a new infrastructure (i.e. construction of a new sanitary block rather than rehabilitating).

Assumptions: In the maintenance costs of infrastructures, you just need to consider the regular maintenance costs (i.e. replacement of components or spare parts), not the cost of mayor renovations/rehabilitations at the end of the lifespan of the whole infrastructure.

Inflation will be considered for the global calculations, but you don't need to provide any information related to inflation or factor it in your estimates; except if you are using old unit costs that you will need to adjust to actual costs in 2020.

Please use the "comments" section to include any additional information that you consider relevant or to clarify any assumptions that you made.

Feel free to reach us if you have any questions in relation to this form.

Figure S1. JMP monitoring definition for basic water services

2.1.1 Basic water services

*Definition: Proportion of health care facilities where the main source of water is an **improved** source, located **on premises**, from which water is **available**.*

Element	Monitoring definition
improved	Improved water sources are those which, by nature of their design and construction, have the potential to deliver safe water. Improved sources include: piped water, boreholes or tubewells, protected dug wells, protected springs, rainwater, and packaged or delivered water. Unimproved sources include unprotected dug wells or springs and surface water (e.g. lake, river, stream, pond, canals, irrigation ditches).
on premises	Water is accessed within buildings, or within the facility grounds.
available	Water from the main water source is available on the day of the survey or questionnaire.

Figure S2. JMP monitoring definition for basic sanitation services

2.1.2 Basic sanitation services

*Definition: Proportion of health care facilities with **improved** and **usable** sanitation facilities, with at least one toilet **dedicated for staff**, at least one **sex-separated toilet** with **menstrual hygiene facilities**, and at least one toilet **accessible for users with limited mobility**.*

Element	Monitoring definition
improved	Improved sanitation facilities are those designed to hygienically separate excreta from human contact. Improved sanitation facilities are those designed to hygienically separate excreta from human contact. Improved facilities include: flush/pour flush to piped sewer system, septic tanks or pit latrines; ventilated improved pit latrines, composting toilets or pit latrines with slabs. Unimproved facilities include pit latrines without a slab or platform, hanging latrines, and bucket latrines. For the purpose of this document "toilets" is taken to mean any of these improved facilities.
usable	Toilets are available, functional, and private:<br>• **Available** to patients and staff (toilets are on premises, doors are unlocked or a key is available at all times)<br>• **Functional** (the toilet is not broken, the toilet hole is not blocked, there should be no cracks or leaks in the toilet structure and water is available for flush/pour-flush toilets), and<br>• **Private** (there are closable doors that can be locked from the inside and no large gaps or holes in the structure) on the day of the survey or questionnaire.
dedicated for staff	There are separate toilet facilities dedicated for patient and staff use.
sex-separated with menstrual hygiene facilities	At least one toilet is separated for use by women/girls, and has a bin with a lid on it and/or water and soap available in a private space for washing.
accessible for users with limited mobility	Toilets are considered accessible if they meet relevant national or local standards. In the absence of such standards, toilets should be accessible without stairs or steps, have handrails for support attached either to the floor or sidewalls, a door which is at least 80 cm wide, and the door handle and seat within reach of people using wheelchairs or crutches/sticks.[16]

Figure S3. JMP monitoring definition for basic hygiene services

2.1.3 Basic hygiene services

*Definition: Proportion of health care facilities with **functional hand hygiene facilities** available at one or more **points of care** and **within 5 metres of toilets***

Element	Monitoring definition
hand hygiene facilities	A hand hygiene facility is any device that enables staff and patients to clean their hands effectively, such as a sink with tap, water tank with tap, bucket with tap or other similar device. Alcohol based hand rub dispensers are also hand hygiene facilities, whether they are fixed or portable.
functional	To be considered functional, hand hygiene facilities at points of care must have either alcohol based hand rub, or soap and water. If alcohol-based hand rub is used, health care staff may carry a dispenser around between points of care.<br><br>To be considered functional, hand hygiene facilities at toilets must have soap and water available within 5 m of toilets. Alcohol-based rub is not considered adequate for hand hygiene at toilet as it does not remove faecal matter from hands.<br><br>Chlorinated water (a prepared solution of chlorine suspended in water) is not considered an adequate substitute for soap and water, or for alcohol based hand rub.
points of care	Points of care are any location in the health care facility where care or treatment is delivered (e.g. consultation/exam rooms).
within 5 m of toilets	Hand hygiene facilities at toilets must be located no more than 5 metres from the toilets.

Figure S4. JMP monitoring definition for basic health care waste management services

2.1.4 Basic health care waste management services

*Definition: Proportion of health care facilities where waste is **safely segregated** in consultation areas and sharps and infectious wastes are **treated and disposed of safely.***

Element	Monitoring definition
safely segregated in consultation area	At least three clearly labelled or colour coded bins should be in place to separate (1) sharps waste[17], (2) infectious waste[18], and (3) non-infectious general waste. Bins should be no more than three quarters (75%) full, and each bin should not contain waste other than that corresponding to its label. Bins should be appropriate to the type of waste they are to contain; sharps containers should be puncture-proof and others should be leak-proof. Bins for sharps waste and infectious waste should have lids. Consultation areas are rooms or areas within the health care facility where care or treatment is delivered.
treated and disposed of safely	Safe treatment and disposal methods include incineration, autoclaving, and burial in a lined, protected pit. Wastes may also be collected and transported off-site for medical waste treatment and disposal.

Figure S5. JMP monitoring definition for basic environmental cleaning services

2.1.5 Basic environmental cleaning practices

*Definition: Proportion of health care facilities which have **protocols for cleaning**, and **staff with cleaning responsibilities** have all received **training** on cleaning procedures.*

Element	Monitoring definition
protocols for cleaning	Protocols should include:<br>• step-by-step techniques for specific tasks, such as cleaning a floor, cleaning a sink, cleaning a spillage of blood or body fluids<br>• a cleaning roster or schedule specifying the frequency at which cleaning tasks should be performed
staff with cleaning responsibilities	Includes non-health care providers, such as cleaners, whose tasks include cleaning, as well as health care providers who, in addition to their clinical and patient care duties, are responsible for cleaning
training	Training refers to structured training plans or programs led by a trainer or appropriately qualified supervisor.

Given that this study does not constitute human subjects research, no formal ethics approval was sought. Nonetheless, data was collected following the principles of informed consent, voluntary participation, confidentiality, and anonymity.

The UNICEF Chief of WASH led the data collection process in each country, sometimes in collaboration with government or WHO representatives, or both. The process of data compilation varied from country to country. In most countries, the data collection instrument was populated jointly by staff members of UNICEF and the Ministry of Health, and, where possible, average costs were derived using data from project implementation records or more robust financial data systems. Elsewhere, a stakeholder committee was formed to gather the data, incorporating civil society organizations and other partners into the process. Finally, in some countries UNICEF staff members filled in the instrument based on their implementation experiences. Per their comments, respondents drew on diverse data sources to populate the instrument, including but not limited to national WASH plans and cost norms, project implementation databases, government and partner budgets, and consultations with key informants. A few respondents provided budgeted amounts for recurrent costs, noting a lack of basis to estimate normative costs based on basic service definitions. Individuals who participated in data collection and consented to being named are acknowledged in the main paper. A small number of government officials only agreed to share data on the conditions that country-specific cost estimates would not be published and that their involvement would remain anonymous. No individual is acknowledged in the main paper without their expressed written consent.

Two of the study authors (MC and SM) reviewed all submitted data and sought clarification and additional detail, as needed, from respondents through email communication facilitated by UNICEF. This communication also helped to explain apparent outliers, such as values driven by high construction costs in especially remote or geologically challenging settings. In some cases, adjustments were made to submitted data to increase comparability across countries. For example, multiple respondents excluded from their quantitative submissions drilling costs for on-premises water sources but noted in their qualitative comments the additional costs associated with drilling. In these cases, the drilling costs were incorporated into the per-facility capital costs for on-premises water sources.

There are several possible drivers of cross-country cost variation (see table 2 in the main paper), including differences in local material and labour costs, availability of domestically produced versus imported infrastructure such as pumps and autoclaves, cost premiums to account for implementation in conflict-affected and remote areas, and locally adapted or defined service standards and practices. Additionally, some of the largest variation with single cost categories likely reflects differences in terrain; for instance, all else equal, drilling costs should be lower in the riverbeds of the Greater Mekong Region than in the basalt rock found throughout the Horn of Africa.

Respondents were directed to account for within-country variability when providing average costs, but there is no guarantee that they fully did so. Moreover, differentiating between hospitals and non-hospitals is too coarse to capture the diversity of facility types and configurations in any country, and the different WASH- and waste management–related investments these may require. Likewise, the urban-rural distinction is too crude in countries with large and varied peri-urban settings where costs may be meaningfully different from more purely urban or rural areas. Countries also differ in their technology mix, and many more technologies have the potential to meet basic service standards than those explicitly included in the cost survey.[6] It was not always clear how respondents dealt with variation within specific cost categories. It was assumed that the cost data provided represented the value of investments needed for the average, across all conditions, within a category. However, some respondents provided additional information in the survey comments, or in follow-up correspondence, which reflected an exclusion of outliers from the averages. In these cases, the average costs were adjusted accordingly.[d]

Missing data

UNICEF did not manage to collect cost information for six LDCs, and several others' submissions were incomplete. A previous global WASH costing exercise favoured an imputation method based on adjusting prices from an economically similar country for differences in per-capita gross domestic product expressed at purchasing power parity.[7] However, exploratory analysis of the cost data from UNICEF's survey failed to reveal any systematic

[d] For example, one response noted that consultations had been conducted with seven regions, and it was estimated that average costs would be 40% higher for the roughly 30% of facilities in challenging environments, such as remote or flood-prone areas. The authors computed a weighted average cost based on these details.

relationship between per-facility costs and potential correlates of cross-country price variation, such as per capita national income or baseline coverage levels. More elaborate econometric methods were also ruled out both due to practical constraints and the prospect that predictive models for 34 different cost variables (17 capital and 17 recurrent) based on data from fewer than 40 countries would be undermined by uncertainty and bias.

Consequently, median per-facility costs were applied when per-facility capital cost estimates were missing. Median values were favoured over arithmetic means because there were notable outliers for most indicators, often attributable to country-specific considerations, explained by survey respondents in their comments or subsequent email communications. The large number of LDCs in Africa allowed for the application of regional medians for missing values in countries located in UNICEF's two operational regions in sub-Saharan Africa (Eastern and Southern Africa; West and Central Africa). For example, no per-facility cost data were received for Niger, so for each capital cost indicator needed, the median value of per-facility capital costs among other LDCs in West and Central Africa was applied. All-LDC medians were applied to countries in other regions (see table S1).[e] In general, fewer countries reported recurrent costs than capital costs, so the imputation of missing recurrent values used the ratio of recurrent to capital costs where both were reported. Thus, the regional or all-LDC median ratio of recurrent to capital costs was applied to impute missing recurrent costs.

Finally, cost values were extrapolated for on-premises water sources at hospitals. These were not solicited by the per-facility cost survey, but a significant number of LDC hospitals are not connected to piped water sources.[f] One of the survey respondents included in their comments that the costs associated with on-premises water sources were roughly 40% greater in hospitals than non-hospitals in their country. Based on this, for each country, the capital and recurrent costs for on-premises water sources for hospitals was estimated to be 1.4 times that country's costs for non-hospitals.

[e] For each cost indicator, the median value was determined only from countries with data for that indicator. The number of countries for which the per-facility cost survey collected data is reported for each capital and recurrent cost indicator in Table 2 of the main paper.

[f] In 17 of the 27 LDCs for which data were available, at least one quarter of hospitals had a non-piped water source.

Section 4: Number of facilities

Extensive internet searches were conducted using Google and Bing to identify the most recently published country-specific information regarding facility quantities and types. The objective was to find an official facility census or other government documentation for each country, or to find other publications (primarily grey literature) containing this information. Numerous search terms and combinations were employed, including country names and phrases such as "health system overview," "health facilities," "health facility census," and similar. Searches were conducted in English, French, and Portuguese. Up to five pages of search results were reviewed until a suitable source was identified. When this initial search strategy did not yield adequate results, manual searches were conducted of the websites of health ministries, development partners such as WHO, the World Bank, USAID and its implementing partners, and WHO's Health Resources Availability Monitoring System (HeRAMS) (herams.org). When multiple sources were identified for the same country, preference was given to whichever contained more recent data, provided its accounting for facilities appeared to be at least as complete as in other sources. When no recent data could be found, outreach was conducted to individuals familiar with the country's health system.

Providers described as "parapublic" or "mixed" were considered public. When clearly identified, most private facilities were removed from the counts and separately tabulated. These included for-profit facilities and those owned and operated by nongovernmental, not-for-profit, and faith-based organizations. However, some privately owned facilities were retained in the counts in two countries due to their considerable inclusion in government planning or financing (or both): facilities owned by the Christian Health Association of Malawi (CHAM) in Malawi and not-for-profit–owned Centres de Santé Communitaires in Mali. When there was no mention of the private sector, it was assumed that the number of health facilities listed in government and development agency documents pertained only to the public sector. Similarly, when some facilities were stratified by private and public ownership by a source, those facilities not designated as either were assumed to be public. If a source quantified the share of all facilities that were privately owned, the published percentage was applied uniformly to all facility types.

The public health facilities identified were filtered to exclude several types, including those acting as retailers (e.g., pharmacies), or those exclusively providing non-patient-facing services (e.g., laboratories) or auxiliary or allied health services (e.g., dental clinics, school clinics). Mobile clinics and other non-fixed/temporary facilities were also excluded. All other facilities described or implied to be part of the public health system were retained, including military establishments and recognized practitioners of traditional medicine, where relevant. All existing facilities were included regardless of operational status.[g] No projections were made of expected changes to facility counts between the time of the study and 2030.

The resulting public health facilities were then sorted into four profiles: urban hospitals, urban non-hospitals, rural hospitals, and rural non-hospitals (tables S4 and S5). A portion of published sources provided sufficiently detailed information to assist sorting, such as tables disaggregating facility types across settings, "urban" or "rural" being included in names of facility types, or qualitative information about the concentration of certain facility types in urban or rural areas. For some countries, additional insight was gleaned from email communication with respondents to the cost survey. When sources lacked these details, assumptions guided the sorting as follows:

- Where there were multiple hospital types, a determination was made about each type. Those that were considered likely to be referral facilities serving geographies larger than an individual district (e.g., regional, national, or specialized hospitals) were all assumed to be in urban areas;
- Other types (e.g., district, municipal, or primary hospitals) were distributed between urban and rural settings in proportion to the country's existing level of urbanization, such that the share of these facilities categorized as urban was equal to the share of the population living in urban areas,[3] with the remainder categorized as rural. This approach is similar to that applied for other global price tags for health (Hanssen O, personal communication);
- Where there was only one hospital type, and the country source made no indication that they were all central or referral facilities, they were sorted based on urbanization levels; and
- Non-hospitals were also sorted based on urbanization levels.

[g] This may have led to overcounting facilities in some countries depending on the respective government's plans for non-operational facilities (e.g., rehabilitation vs. decommissioning). The source for one country (Angola) explicitly only included the number facilities considered "functional."

Table S4. Exclusions and sorting of documented facility types, by country[h]

Country	Setting assignment	Hospitals (secondary, tertiary, and highly specialized facilities)	Non-hospitals (health centres, clinics, posts, and similar)
Afghanistan	Excluded	··	Mobile Clinic
	Urban	Provincial Hospital, Regional Hospital, Special Hospital	··
	Rural	··	··
	Split by urbanization	District Hospital	Sub-centre, Basic Health Centre, Comprehensive Health Centre, Other, Health Post
Angola	Excluded	··	··
	Urban	Provincial Hospital, Central/National Hospital	Specialized Health Center
	Rural	··	··
	Split by urbanization	Municipal Hospital	Heal Post, Health Center, Maternal & Child Health Center, Non-classified Health Facility
Bangladesh	Excluded	Dental College Hospital	Private Facility, School Health Clinic
	Urban	Leprosy Hospital, Infectious Disease Hospital, Hospital of Alternative Medicine, Medical College Hospital, Specialized Hospital, Specialized Health Center (Tertiary Level), Specialty Postgraduate Institute and Hospital, Trauma Center, Other Hospital, Chest Hospital, Health Office (Tertiary Level)	Urban Dispensary
	Rural	··	··
	Split by urbanization	Primary Care Hospital (Upazila and Below), District-level Hospital	Health Office (Secondary Level), Chest Disease Clinic, Health Complex, Outdoor Only/Out-Patient Only Primary Health Facility (Upazila and Below), Community Clinic
Benin	Excluded	··	Private Facility, Officine
	Urban	CHD (Centre Hospitalier de Référence de District), Hôpitaux Nationaux, Autres Hôpitaux	··
	Rural	··	··
	Split by urbanization	HZ (Hôpital de Zone)	CS (Centre de Santé), Dispensaire Seul, Maternité Seule
Bhutan	Excluded	··	Outreach Clinic without Shed
	Urban	··	··
	Rural	··	··
	Split by urbanization	Hospital	Primary Health Center, Sub-post, Outreach Clinic with Shed, Thromde Health Center, Health Information and Service Center (HISC)
Burkina Faso	Excluded	··	Officines et Dépôts Privés
	Urban	CHR (Centre Hospitalier Regional), CHU (Centre Hospitalier Universitaire)	··
	Rural	··	··
	Split by urbanization	CMA (Centre Médical Avec Antenne Chirurgicale)	Maternité Isolée, Centre de Santé et de Promotion Sociale, Centre Médical, Dispensaire
Burundi	Excluded	Private Hospital	Private Facility
	Urban	··	··
	Rural	··	··
	Split by urbanization	Public Hospital	Health Center

[h] See references in the Country column of Table S5 for the source of each country's facility types and counts.

Cambodia	Excluded	Private Hospital	Private Facility
	Urban	National Hospital	··
	Rural	··	··
	Split by urbanization	District-based Referral Hospital, Municipal and Provincial Referaral Hospital	Health Post, HC (Health Center)
Central African Republic	Excluded	··	Private Facility
	Urban	Regional University Hospital, Central Hospital, Secondary Hospital	··
	Rural	··	··
	Split by urbanization	District Hospital	Health Center, Health Post
Chad	Excluded	National Blood Transfusion Center, Sub-national Vaccine Depot, National Center for Devicing/Rehabilitation, Central Store, Pharmaceutical Purchasing Plant, Private Hospital	Pharmacy, Private Facility
	Urban	Hôpitaux Régionaux, Hôpitaux Nationaux, Hôpital de la Mére et de L'enfant, Centre National de Traitement des Fistules	··
	Rural	··	··
	Split by urbanization	Hôpitaux des district	CS (Centre de Santé)
Comoros	Excluded	··	Private Facility
	Urban	National Referral Hospital, Regional Hospital	··
	Rural	··	··
	Split by urbanization	··	Health Post, District Health Center
Democratic Republic of Congo	Excluded	Private Hospital	Private Facility
	Urban	··	··
	Rural	··	··
	Split by urbanization	Hospital Structure	Health Center
Djibouti	Excluded	Private Hospital	Private Facility
	Urban	Hôpitaux de Référence, Hôpital Régional, Hôpital de Le Secteur Parapublic	Centres de Santé et Polycliniques en Zone Urbaine, Centres Spécialisés, Centres de Le Secteur Parapublic
	Rural	··	Postes de Santé en Zone Rurale
	Split by urbanization	Centres Médicaux Hospitaliers (CMH)	
Eritrea	Excluded	National Blood Transfusion Center	Laboratory, Regional Blood Transfusion Center
	Urban	··	··
	Rural	··	··
	Split by urbanization	Hospital	Health Center, Health Station
Ethiopia	Excluded	··	··
	Urban	General Hospital, Referral Hospital	··
	Rural	··	··
	Split by urbanization	Primary Hospital	Health Post, Health Center
Gambia	Excluded	Private Hospital	Private Facility
	Urban	General Hospital, Teaching and Specialty Hospital	··
	Rural	··	··
	Split by urbanization	District Hospital	Service Clinic, Primary Healthcare Village Post, Reproductive and Child Health Center, Community Clinic, Minor Health Center, Major Health Center
Guinea	Excluded	Private Hospital	Private Facility
	Urban	Hôpitaux Régionaux, Hôpitaux Nationaux	··
	Rural	··	··
	Split by urbanization	Centres Médicaux Communaux et Hôpitaux Préfectoraux	Postes de Santé, Centres de Santé, Centres de Santé Améliorés

Guinea-Bissau	Excluded		
	Urban	Regional Hospital, National Hospital	··
	Rural	··	··
	Split by urbanization	··	Health Centre, MCH Centre
Haiti	Excluded	Private Hospital	Private Facility
	Urban	··	··
	Rural	··	··
	Split by urbanization	Hôpital	Centre de Santé Avec Lit, Centre de Santé Sans Lit, Dispensaire
Kiribati	Excluded	··	··
	Urban	··	··
	Rural	··	··
	Split by urbanization	Hospital	Health Center, Village Clinic
Lao People's Democratic Republic	Excluded		Private Facility
	Urban	Army Hospital, Police Hospital, Provincial Hospital, Regional Hospital, Curative Centre at Central Level, Central Hospital	··
	Rural	··	··
	Split by urbanization	District Hospital	Health Centre
Lesotho	Excluded	··	Private Facility
	Urban	Referral Hospital, Specialized Hospital	··
	Rural	··	··
	Split by urbanization	District Hospital	Health Centre, Nurse Clinic, Filter Clinic
Liberia	Excluded	Private Hospital	Pharmacy, Private Facility
	Urban	··	··
	Rural	··	··
	Split by urbanization	Hospital	Health Center, Clinic
Madagascar	Excluded	Private Hospital	Private Facility
	Urban	CHU (Centre Hospitalier Universitaire), HMP (Hopitaly Manara Penitra), CHRR (Centre Hospitalier de Référence Régionaux)	··
	Rural	··	··
	Split by urbanization	CHD (Centre Hospitalier Départemental)	CSB (Centre de Santé de Base),
Malawi	Excluded	Private Hospital	Private Facility
	Urban	··	··
	Rural	··	··
	Split by urbanization	Hospital	Dispensary, Health Centre, Health Post, Outreach, Village Clinic
Mali	Excluded	··	··
	Urban	Regional Hospital	··
	Rural	··	··
	Split by urbanization	··	Community Health Center, First Referral Facility
Mauritania	Excluded	··	Private Facility
	Urban	Regional and National Hospital	··
	Rural	··	··
	Split by urbanization	··	Health Post, Health Center
Mozambique	Excluded	Private Hospital	Private Facility
	Urban	Provincial Hospital, Central, Military and Specialized Hospital	··
	Rural	··	··
	Split by urbanization	General, Rural and District Hospital	Urban and Rural Health Center and Health Post
Myanmar	Excluded	··	School health Clinic
	Urban	··	··

	Rural	··	Rural Health Center
	Split by urbanization	Hospital, Traditional Medicine Hospital	Primary and Secondary Health Center, Traditional Medicine Clinic, Maternal and Child Health Clinic
Nepal	Excluded	··	Private Facility
	Urban	Urban National/Zonal Hospital, Urban District Hospital	Urban PHC Center, Urban Health Post, Urban Other
	Rural	··	Rural PHC Center, Rural Health Post, Rural Other
	Split by urbanization	··	··
Niger	Excluded	Private Hospital	Private Facility
	Urban	Maternité de Reference, Centre Hospitalier Régional (CHR), Hôpital des Armées, Hôpital National	··
	Rural	··	··
	Split by urbanization	Hôpital de District	Centre de Santé Intégré de Type I, Centre de Santé Intégré de Type II, Case de santé
Rwanda	Excluded	Private Hospital	Private Facility
	Urban	National Referral Hospital, Provincial Hospital	··
	Rural	··	··
	Split by urbanization	District Hospital	Health Center, Prison Clinic, Health Post
São Tomé and Príncipe	Excluded	··	··
	Urban	Hospital Central, Hospital Regional	··
	Rural	··	··
	Split by urbanization	··	Centros de Saúde, Postos de Saúde, Centros de Saúde Reprodutiva, Postos Comunitários de Saúde
Senegal	Excluded	Private Hospital	Private Facility
	Urban	EPS hospitalier niveau 2, EPS hospitalier niveau 3	··
	Rural	··	··
	Split by urbanization	EPS hospitalier niveau 1	Case de Santé, Poste de Santé, Centre de santé, EPS non hospitalier
Sierra Leone	Excluded	Private Hospital	Private Facility
	Urban	Primary Tertiary Hospital	··
	Rural	··	··
	Split by urbanization	Hospital	Clinic, Community Health Center, Community Health Post, Maternal and Child Health Post
Solomon Islands	Excluded	··	··
	Urban	Hospital	··
	Rural	··	Nurse Aid Post, Rural Health Center, Area Health Center
	Split by urbanization	··	
Somalia	Excluded	Private Hospital	Mobile Clinic, Private Facility
	Urban	··	··
	Rural	··	··
	Split by urbanization	Hospital	Referral Health Center, Health Center, Primary Health Unit, TB Center, Nutrition Center, Other
South Sudan	Excluded	··	Boma Health Initiative
	Urban	··	··
	Rural	··	··
	Split by urbanization	Hospital	Health Facility, PHCU (Primary Healthcare Unit), PHCC (Primary Healthcare Clinic)
Sudan	Excluded	Private Hospital	Private Facility

	Urban	General State Hospital, Specialized State Hospital, Specialized Federal Hospital, Police Hospital, Military Hospital, Other Hospital	Urban Health Center
	Rural	Rural State Hospital	Rural Health Center
	Split by urbanization	··	Basic Health Unit
United Republic of Tanzania	Excluded	··	··
	Urban	··	··
	Rural	··	··
	Split by urbanization	Hospital	Health Center, Dispensary, Maternity/Nursing Home, Other
Timor-Leste	Excluded	··	SISca (Servisu Integrado Sude Communita), Private Facility
	Urban	··	··
	Rural	··	··
	Split by urbanization	Hospital	Health Post, Health Center
Togo	Excluded	··	··
	Urban	Hopital II, Hopital specialisé, CHR (Centre Hospitalier Regional), CHU (Centre Hospitalier Universitaire)	··
	Rural	··	··
	Split by urbanization	Hopital I	Autre, USP (unité de soins périphérique) I, USP (unité de soins périphérique) II
Tuvalu	Excluded	··	··
	Urban	Hospital	··
	Rural	··	··
	Split by urbanization	··	Health Clinic, Health Center
Uganda	Excluded	Private Hospital	Private Facility
	Urban	National Referral Hospital, Referral Hospital, Regional Referral Hospital	Special Clinic
	Rural	··	··
	Split by urbanization	Hospital	Clinic, Health Center II, Health Center III, Health Center IV
Yemen	Excluded	Private Hospital	Private Facility
	Urban	General Hospital, Referral Hospital	
	Rural	··	··
	Split by urbanization	District Hospital	Health Center, Primary Health Care Unit, Health Unit
Zambia	Excluded	Private Hospital	Private Facility
	Urban	Hospital level 2, Hospital Level 3, Police Hospital, Military Hospital	Urban Health Center
	Rural	··	Rural Health Center
	Split by urbanization	Hospital Level 1	Zonal Health Center, Border Health Post, Health Post, Hospital Affiliated Health Center

Table S5. Facility counts by profile and country

Country	Urban Hospitals	Urban Non-hospitals	Rural Hospitals	Rural Non-hospitals	Private Facilities[*]
Afghanistan[8]	88	5216	62	14824	..
Angola[9]	148	1500	55	733	..
Bangladesh[10]	291	5944	336	9569	14850
Benin[11]	44	468	15	498	121
Bhutan[12]	21	303	28	413	..
Burkina Faso[13]	29	690	34	1564	..
Burundi[14]	6	78	39	488	479
Cambodia[15]	32	302	70	946	7626
Central African Republic[16]	36	343	16	469	155
Chad[17]	21	314	27	1020	436
Comoros[18]	4	26	0	64	7
Democratic Republic of the Congo[19]	116	3881	138	4623	1504
Djibouti[20]	9	27	1	41	26
Eritrea[21]	12	99	16	140	..
Ethiopia[22]	143	4137	128	14931	..
The Gambia (Republic of)[23]	10	476	1	285	39
Guinea[24]	22	494	21	846	7
Guinea Bissau[25]	6	61	0	76	..
Haiti[26]	36	265	27	199	378
Kiribati[27]	2	58	2	47	..
Lao People's Democratic Republic[28]	111	383	87	672	1028
Lesotho[29]	8	75	13	182	48
Liberia[30]	14	280	13	258	160
Madagascar[31]	78	1025	53	1635	713
Malawi[32]	14	1610	69	7631	174
Mali[33]	12	596	0	761	..
Mauritania[34]	26	445	0	360	138
Mozambique[35]	33	579	32	984	224
Myanmar[36]	333	209	737	2147	..
Nepal[37,†]	125	2834	0	1904	2071
Niger[38]	18	575	28	2886	318
Rwanda[39]	18	245	30	1162	280
São Tomé and Príncipe[40]	2	45	0	15	..
Senegal[41]	30	1777	5	1915	1508
Sierra Leone[42]	12	503	12	669	72
Solomon Islands[‡]	11	0	0	319	..
Somalia[43]	52	663	61	773	31
South Sudan[44]	22	368	89	1456	..
Sudan[45]	246	1761	269	3756	345
United Republic of Tanzania[46]	104	3103	190	5707	..
Timor-Leste[47]	2	113	4	248	30
Togo[48]	66	480	37	641	..
Tuvalu[49]	1	6	0	4	..
Uganda[50]	26	769	55	2282	3804
Yemen[51]	125	1448	114	2372	2970
Zambia[52]	62	904	42	1789	284
**Total**	**2,628**	**45,478**	**2,955**	**94,303**	**39,826**

[*]Sources that provided private facility data may be incomplete for the private sector. Numbers noted here aggregate all facility types, both hospitals and non-hospitals, except those incorporated in the public facility counts as described in the text above. Otherwise, private sector facilities were not included in the analysis.

†Nepal data was sorted with information directly from country representatives rather than by the assumptions in Table S2.

[‡]Facility data for Solomon Islands were sourced informally from the Ministry of Health and Medical Services (Tevera A, UNICEF Solomon Islands, personal communication).

Section 5: Quantifying needs

WASH needs were based on 2019 coverage data published by the JMP, which has published estimates of country, regional and global progress toward WASH objectives since 1990. The methods underpinning the JMP's estimates for health care facilities are documented elsewhere.[5,6]

The JMP defines service ladders with four rungs for each WASH service: Advanced, Basic, Limited, and No service. Advanced service levels are defined at the country level, not globally, and thus were not included in this multi-country analysis. In this study, the coverage target was that all facilities would meet at least the *Basic* service level by 2030. Estimating costs for the portion of facilities in the *No* service category was straightforward: they were assigned the full per-facility costs for the relevant service(s). In contrast, estimating costs for facilities with *Limited* services would need investments smaller than the full, per-facility costs for the relevant service. However, to best reflect the variation within this category, the estimate of additional costs required additional disaggregation, due to the diversity of on-the-ground realities of facilities at that level, and because the definition of *Limited* differed by service.

Limited service level for water and sanitation

For water, a facility can fall short of the *Basic* service level for as little as a broken pipe and as much as the lack of an on-premises water source. Similarly, for sanitation, a facility might not meet the basic service levels due to the lack of waste bins for menstrual hygiene products or there only being one improved toilet but no sex separation or accommodation for those with limited mobility. Consequently, data were sought that would allow division of the *Limited* category between facilities requiring investment of comparable magnitude to those at the *No* service level (full investment) and those whose needs were somewhat less (reduced investment). Other JMP indicators allowed for identification of the portion of facilities with or without the core water and sanitation infrastructure that was assumed to drive the largest portion of capital costs: an improved, on-premises water source and an improved, usable sanitation facility. Facilities with those assets were then assumed only to require reduced capital investment, while facilities lacking those assets were assumed to require the same capital investment as facilities at the *No* service level.

In the absence of more granular cost or needs data, consultations were conducted with an expert steering group to determine what share of the per-facility capital costs should be applied to the facilities requiring reduced investment for water or sanitation (table S6). The steering group determined that the main estimates should assume that reduced investments would require 50% of the per-facility capital costs. In other words, it was assumed that, on average, sub-standard facilities that already had the core water or sanitation infrastructure would require half as much upfront investment as facilities at or above the *Basic* service level. Recognizing considerable uncertainty in this assumption, the steering group recommended producing two additional cost estimates, lower and upper, using 15% and 85% of the per-facility capital costs, respectively, for the facilities that required reduced investments. Additionally, the steering group agreed that all in-need facilities—those requiring either full or reduced investment—required 100% of the per-facility recurrent costs for water and sanitation. Therefore, these were not varied in the lower and upper estimates.

Limited service level for hygiene and waste management

For hygiene, a facility is at the *Limited* service level if hand hygiene facilities are available at points of care or within five metres of sanitation facilities, but not at both. There are JMP indicators for both locations within facilities, allowing separate calculations of how many facilities lack hygiene at points of care and in proximity to toilets. These two indicators together capture all facilities at the *Limited* and *No* service levels. Similarly, the *Limited* service level for waste management involves some level of waste segregation, treatment, and disposal, and the JMP has separate indicators for (i) segregation and (ii) treatment and disposal.

In the absence of more granular cost or needs data, assumptions were made, again informed by consultations with the steering group and additional experts, regarding the portion of per-facility costs applied to the reduced investment categories (table S7). For hygiene, non-hospitals were assumed to have an equal number of points of care and sanitation facilities, so a "reduced investment" in this category meant that 50% of the per-facility costs for sanitation facilities and points of care were used. Hospitals were assumed to have three times as many points of care

as sanitation facilities, so "reduced investments" for this type of facility were assumed to be equivalent to 75% and 25% of the per-facility costs for points of care and toilets, respectively. For waste management, treatment and disposal was assumed to be much more capital intensive than segregation (incinerators and autoclaves cost more than waste bins), while the reverse was assumed for recurrent needs. Thus, for facilities lacking waste segregation, the "reduced investment" was estimated as being equal to 25% of the per-facility capital costs and 75% of the per-facility recurrent costs for waste management. Similarly, for facilities lacking waste treatment and disposal, the "reduced investment" was estimated as being equal to 75% of the capital and 25% of the recurrent costs of the full per-facility costs for waste management (Pieper U, personal communication).

Table S6. Quantifying needs for water and sanitation

**Investment need**	**Definition for Water**	**Definition for Sanitation**	**Share of per-facility capital costs applied**	**Share of per-facility recurrent costs applied**
Requiring no investment	Share of facilities meeting at least the *Basic* service level	Share of facilities meeting at least the *Basic* service level	0%	0%
Requiring reduced investment	Share of facilities with an improved, on-premises water source but falling short of the *Basic* service level (subset of facilities at the *Limited* service level)	Share of facilities with improved, usable sanitation facilities but falling short of the *Basic* service level	50% for baseline estimates, 15% for lower estimates, and 85% for upper estimates	100%
Requiring full investment	Share of facilities without an improved, on-premises water source (subset of facilities at the *Limited* service level plus all facilities at the *No* service level)	Share of facilities without improved, usable sanitation facilities (subset of facilities at the *Limited* service level plus all facilities at the *No* service level)	100%	100%

Table S7. Quantifying needs for hygiene and waste management

**Investment need**	**Definition for Hygiene**	**Share of per-facility hygiene costs needed**	**Definition for Waste management**	**Share of per-facility waste management costs needed**
Requiring no investment	Share of facilities meeting at least the *Basic* service level	0%	Share of facilities meeting at least the *Basic* service level	0%
Requiring reduced investment – category 1	*Points of care:* Share of facilities lacking hand hygiene facilities with water and soap and/or alcohol-based hand rub at points of care	For hospitals, 75% of capital and 75% of recurrent. For non-hospitals, 50% of capital and 50% of recurrent.	*Segregation:* Share of facilities failing to safely segregate waste into at least three bins	25% of capital and 75% of recurrent
Requiring reduced investment – category 2	*Sanitation facilities:* Share of facilities lacking hand hygiene facilities with water and soap and/or alcohol-based hand rub within five metres of toilets	For hospitals, 25% of capital and 25% of recurrent. For non-hospitals, 50% of capital and 50% of recurrent.	*Treatment and disposal:* Share of facilities failing to safely treat and dispose of sharps and medical waste	75% of capital and 25% of recurrent

Matching JMP indicators to facility profiles

The JMP publishes estimates of national service coverage levels for WASH in health care facilities, broken down by several stratifications: urban and rural facilities, hospital and non-hospital facilities, and government and non-government facilities. The last stratification was not used in this analysis given the exclusion of private facilities in the estimation of country-level resource needs. These, however, are not available for combinations of these stratifications, and so sorting rules were defined to match coverage indicators to the four facility profiles used in the cost analysis. Preference was given to either of the JMP strata matching the facility type (e.g., for rural non-hospitals, the JMP estimate for rural facilities was applied if available; if not, the estimate for non-hospitals was applied).

When the JMP lacked estimates for either of the two preferred strata for a particular facility profile, other values for the same country were used as proxies. Candidate strata were ranked based on correlation analysis using countries

with data for multiple strata (table S8). For any given stratum, the Kendall Tau correlation coefficient was computed utilizing each other stratum to determine the preference order. Any stratum yielding a coefficient of at least 0.5 was considered to be a valid proxy and ordered from greatest to least correlation coefficient.[i] When repeated with Spearman's Rho and Pearson's correlation tests, the results were broadly the same. All correlation analysis was conducted in R using the "cor" function and restricting analysis to complete observations.

If no data were available and all candidate proxies failed to pass the correlation test, the LDC average[j] from the corresponding first choice stratum was applied.[k] Some manual adjustments were required when the application of LDC averages led to negative results when one coverage indicator was subtracted from another.[l]

Table S8. Sequence of application for JMP stratified coverage indicators

Service	Facility profile	Rural	Urban	Hospital	Non-hospital	National	LDC average
Water	Urban hospital	DNQ	1	2	DNQ	3	4
Water	Urban non-hospital	4	1	DNQ	2	3	5
Water	Rural hospital	2	DNQ	1	3	4	5
Water	Rural non-hospital	1	DNQ	DNQ	2	3	4
Sanitation	Urban hospital	5	1	2	4	3	6
Sanitation	Urban non-hospital	5	1	3	2	4	6
Sanitation	Rural hospital	2	4	1	5	3	6
Sanitation	Rural non-hospital	1	3	DNQ	2	4	5
Hygiene	Urban hospital	DNQ	1	2	DNQ	3	4
Hygiene	Urban non-hospital	4	1	DNQ	2	3	5
Hygiene	Rural hospital	2	DNQ	1	3	DNQ	4
Hygiene	Rural non-hospital	1	DNQ	DNQ	2	3	4
Waste management	Urban hospital	4	1	2	5	3	6
Waste management	Urban non-hospital	4	1	5	2	3	6
Waste management	Rural hospital	2	3	1	5	4	6
Waste management	Rural non-hospital	1	4	5	2	3	6

DNQ = did not qualify based on the 0.5 correlation coefficient threshold; JMP = Joint Monitoring Programme

Note: Correlation analysis was conducted on a single JMP indicator type for each service: improved and on-premises source for water (*_imop*), improved and usable for sanitation (*_ius*), points of care for hygiene (*_poc*), and segregation for waste management (*_seg*).

[i] Kendall Tau was selected due to the non-parametric nature of the coverage data (bounded by 0% and 100%) and its tendency to yield smaller coefficients than Spearman Rho, the other common correlation coefficient for non-parametric data. The rationale was to favour a more conservative test (i.e., the one from which estimated correlations would be less likely to clear the threshold for proxy validity). Below the threshold of 0.5, it was assumed that a regional or all-LDC average for the same facility type would be superior to a same-country value for a different facility type.

[j] The LDC averages published by the JMP at the time of data collection were based on the 47 countries classified as LDCs prior to Vanuatu's graduation in December 2020. Vanuatu represented only 0·03% of the total population across all LDCs and so did not have any measurable impact on LDC averages (Johnston R, personal communication).

[k] The JMP did not have estimated LDC averages for all strata. For sanitation, the non-hospital value was used when the model sought the urban value, and the national value was used when the model sought the hospital value. Similarly, for hygiene the non-hospital value was used when the model sought the national, urban, or hospital value.

[l] For five countries, application of the LDC average to water coverage led to a negative result, and thus, a value was assigned to *wat_bas* (basic) that was equal to 50% of the value of *wat_imop* (improved and on premises). Similarly, for sanitation coverage, application of the LDC average led to a negative result for one country. In this case, a value was assigned to *san_ius* (improved and usable) that was equivalent to *san_bas* (basic).

Exclusion of environmental cleaning

There are far fewer data for coverage of environmental cleaning than other WASH services. The JMP only had national coverage estimates for four LDCs (Bhutan, Malawi, Niger, and Rwanda) and some stratified estimates for an additional four (Ethiopia, Mali, Mozambique, and Zambia). These countries' combined population was too small for the JMP to impute an all-LDC average.[m] Along with the mismatch between the definition of basic cleaning services and the UNICEF cost data for cleaning, this contributed to the decision to exclude environmental cleaning from the analysis.

[m] The JMP produces estimates for country groupings when data are available for countries containing at least 30% of the grouping's total population.

Section 6: Water and sanitation service assumptions

Although a range of technologies can be deployed to meet the basic service standards for WASH in health care facilities,[6] for water and sanitation, the per-facility cost survey narrowed the options to only two categories each. Many more are used in practice. The resulting database contained separate cost estimates for piped and on-premises water sources and for sewerage- and septic-based sanitation systems. Consequently, assumptions were made about what share of in-need facilities would require costs associated with networked versus other systems for water and sanitation services.

It was assumed that in-need facilities would incur the costs of networked services—piped water and sewerage-linked sanitation—in proportion to the baseline availability of those systems in a country. By extension, all other facilities would be assumed to require the costs for non-networked services (on-premises water sources and septic-based sanitation). The model did not attempt to anticipate increases in the availability of networked infrastructure in the decade ending in 2030, nor did it estimate the more general infrastructure costs that such increases would entail.

Information about the prevalence of networked water and sanitation services came from three sources:

1. In some cases, respondents to the per-facility cost survey included information about the universality or unavailability of certain kinds of services in their country. This information was accepted except under specific circumstances (see next item).
2. Relevant data were extracted from JMP country files, which consolidate information about health infrastructure from a range of nationally representative surveys. For each JMP stratum (national, urban, rural, hospital, and non-hospital), the most recent estimate was sought for the prevalence of piped water and sewerage-linked sanitation in health care facilities. Data were only accepted if the source survey was published after 2010 and considered sufficiently representative to be used in the JMP's own analysis. These data overrode information gleaned from cost survey comments in rare instances of significant disagreement.[n] In total, the estimated prevalence of networked services was found for 34 countries for water and 16 countries for sanitation.
3. When it was not possible to determine the prevalence of service types from either of the first two sources, the shares of households with piped water or sewerage-linked sanitation from the JMP household data[o] for 2017 were used as proxies.[p]

The data on service types were stratified identically to the coverage data, so the same correlation analysis technique was applied to match those strata to the facility profiles in the model (table S9; section 5). The household WASH service data were only stratified between urban and rural settings.

---

[n] If a UNICEF survey respondent indicated that a technology was not available in the country, but data from the WHO/UNICEF JMP country file suggested a prevalence of 10% of greater, the latter prevailed. This occurred for only three of the 92 technology prevalence estimates: sanitation in Bangladesh, sanitation in Haiti, and water in the Solomon Islands.

[o] WHO/UNICEF JMP country files and household data are available at washdata.org.

[p] For Central African Republic and Eritrea, the most recent household data were from 2016 and were applied.

Table S9. Sequence of application of JMP country file prevalence estimates for networked water and sanitation services

Service	Facility profile	Rural	Urban	Hospital	Non-hospital	National	Rural households*	Urban households
Water	Urban hospital	5	1	2	4	3	NA	6
Water	Urban non-hospital	5	1	4	2	3	NA	6
Water	Rural hospital	2	4	1	5	3	6	NA
Water	Rural non-hospital	1	4	DNQ	2	3	5	NA
Sanitation	Urban hospital	5	1	2	4	3	NA	6
Sanitation	Urban non-hospital	5	1	4	2	3	NA	6
Sanitation	Rural hospital	2	5	1	4	3	6	NA
Sanitation	Rural non-hospital	1	4	DNQ	2	3	5	NA

DNQ = did not qualify based on the 0.5 correlation coefficient threshold; JMP = Joint Monitoring Programme; NA = not applicable

*For sanitation in Kiribati, only unstratified household data were available, so the national estimate for households was applied to both rural and urban facilities.

Section 7: Modelling scale-up and asset replacement

The estimates presented are based on a model that assumes a linear, ten-year scale-up, from baseline coverage levels of WASH and waste services in health care facilities to full coverage of the basic service level by 2030. Initial capital investments were assumed to be equally distributed across the ten years, such that 10% of the capital costs—not including replacement costs—were distributed to each year. Due to the urgent need of these investments given their potential contributions to the achievement of multiple SDGs, capital investments would ideally be front-loaded. In fact, the global targets for WASH in health care facilities include 80% coverage by 2025. However, LDCs are among the countries with the greatest resource constraints and least absorptive capacity,[53] raising doubts about the ability of LDC governments and their partners to rapidly finance and build large quantities of assets. Consequently, linear scale-up may be the most ambitious investment trajectory that would be feasible in LDCs.

Each year, additional recurrent costs were estimated in proportion to that year's accumulated capital investments, such that annual recurrent costs were relatively small in 2021 and increased annually through 2030. Finally, because some newly installed assets were not expected to last through 2030 (tables S10 and S11), replacement costs were estimated separately to reflect the need for countries to make additional rounds of capital investments as assets expired. The total capital costs presented in the findings included both the initial capital costs and all replacement costs.

The timing of replacement costs was based on the average expected lifespan of WASH infrastructure. Published studies and technology specifications were reviewed to determine indicative lifespans for WASH technologies deployed in households, communities, health care facilities, and other institutional settings.

Table S10. Average useful lifespans published for WASH technologies

Service	Technology	Setting	Lifespan (Years)
Water	Handpump[54]	Community	18
Water	Rainwater harvesting system[55]	Household, community	10–15
Water	Rainwater harvesting tank[56]	Household	20
Water	Dug well[7]	Household	10
Water	Borehole[7]	Household	20
Water	System with borehole, protected well, and stand posts[57]	Household	20
Water	Pipes[7]	Household	20
Sanitation	Rural traditional pit latrine[7]	Household	2
Sanitation	Eco-San toilet[58]	Community	5–10
Sanitation	Latrine[7]	Household	8
Sanitation	Crestanks WonderLoo Dry Toilet[59]	Household	5–10
Sanitation	Loowatt Toilet[60]	Household	10
Sanitation	Septic Tank, Sewerage, Treatment Facilities[7]	Household	20
Sanitation	Toilets, bidets, urinals[61]	Household	40
Hygiene	Hand washing equipment[7]	Household	1–5*
Hygiene	Tippy Tap[62]	Household	1–2
Hygiene	Raised bucket with tap/outlet[62]	Household	1–2
Hygiene	Two buckets suspended with rope[62]	Household	1–2
Hygiene	Suspended bottle or bag with outlet/hole/pop-up plug[62]	Household	1–2
Hygiene	Sink with tap[62]	Household, community	10
Hygiene	Foot-pump sink[62]	Household, community	1–2
Hygiene	Purpose-built all in one system[62]	Household, institutional	2–4
Hygiene	Free standing water tank with tap(s) or outlet(s)[62]	Community	2–4
Hygiene	Tube with outlets[62]	Community	5–8
Waste Management	De Montfort incinerator[63]	Healthcare	3–5
Waste Management	Steam sterilizer (medical autoclave)[64]	Healthcare	12
Waste Management	Steam sterilizer (medical autoclave) table-top[64]	Healthcare	10
Waste Management	Sharpsology 2 plastic sharps disposal container[65]	Healthcare	5
Waste Management	T4T Mak IV incinerator[66]	School, institutional	5
Waste Management	Solid waste incinerator[67]	Healthcare	10–15

*This range of lifespans is based on multiple technology options that are not detailed in the source.

All the water and sanitation technologies used in the baseline estimates were assumed to have lifespans of at least ten years, so they did not to require replacement during the period from 2021 through 2030. Hygiene technologies in facilities with piped water were also assumed to last at least ten years, given the estimated lifespan of a sink with tap. In all other facilities, new hygiene capital was replaced after two years in the model. For waste management, hospitals were assumed to use sterilization and incineration technologies that were expected to last at least ten years. Non-hospitals were assumed to use De Montfort incinerators or similar technologies, which were expected to be replaced after four years in the model. In the upper estimates, the average expected lifespans of certain water and sanitation assets were shortened due to expected impacts of more frequently occurring extreme weather events, a consequence of climate change (see section 8).

The full value of replacement costs was fed into the model following the year of asset expiration. For example, some facilities lacking piped water received new hygiene assets in year 1 with an expected lifespan of two years, triggering replacement costs in years 3, 5, 7, and 9. Similarly, some non-hospitals received new waste management assets in year 1 with an expected lifespan of four years, triggering replacement costs in years 5 and 9.

Table S11. Average expected lifespans factored into the cost model

Service technology and setting	Lifespan – baseline estimates
Water – piped	>10 years
Water – on-premises	>10 years*
Sanitation – sewerage	>10 years
Sanitation – septic	>10 years*
Hygiene – facility with piped water	>10 years
Hygiene – facility with on-premises water source	2 years
Waste management – hospital	>10 years
Waste management – non-hospital	4 years

*Expected lifespans shortened to 7 years for the upper estimates (see section 8).

Section 8: Sensitivity analysis

Due to uncertainty in several model assumptions, lower and upper estimates were produced by varying selected parameters (table S12). First, alternative assumptions were imposed to address variation within the *Limited* coverage category for water and sanitation services. As detailed in section 5, in the baseline estimates, facilities with major water or sanitation assets—i.e., those with an on-premises and improved water source or improved and usable sanitation facility—but failing to meet the *Basic* service level were assumed to require reduced investment amounting to half as much capital investment as facilities lacking any water or sanitation services. Based on consultation with the expert steering group (see section 1), it was alternatively assumed that these facilities required only 15% of the full per-facility capital cost in the lower estimates, as if they only needed minor upgrades. Similarly, it was assumed these facilities required 85% of the full per-facility capital cost in the upper estimates, as if they required major upgrades or rehabilitation approaching the cost of replacement.

Second, there is inherent uncertainty to any discount rate applied to future costs. The baseline estimates were based on an assumed annual discount rate of 5%. Arguments were considered for other discount rates. For example, methods promoted by both the Global Health Costing Consortium[68] and WHO's Choosing Interventions that are Cost-Effective (CHOICE)[69] include the use of a 3% annual discount rate for costs. In contrast, historical World Bank guidance on the economic evaluation of investments in low- and middle-income countries favoured discount rates greater than 10% due to high costs of capital in those settings.[70]

Discount rates in part represent the opportunity cost of present versus future consumption, a function of expected economic growth.[71] Future growth is unpredictable, particularly in the wake of a major shock, such as that caused by the ongoing global SARS-CoV-2 pandemic. The economic recovery may occur with greater, equal, or lesser speed than expected at global and national levels. Consequently, the lower and upper estimates incorporated alternative discount rates: 8% for the lower estimates, reflecting the possibility of unexpectedly rapid economic growth between 2021 and 2030, and 3% for the upper estimates, reflecting the possibility of slower-than-expected growth during that period. This approach has the additional benefit of aligning with the discount rates applied in the baseline, lower, and upper estimates in previous costing of global WASH targets for households, as defined under SDG 6.[7]

Finally, climate change is increasing the frequency and severity of climate hazards, such as floods and droughts, that can degrade WASH infrastructure.[72] The model incorporated replacement costs into estimated capital costs for those assets with average expected lifespans of less than ten years. For the baseline estimates, these included hygiene assets in health care facilities with non-piped water and waste management assets in non-hospitals (see section 7). Because the relevant climate risks are one-tailed—future climatological conditions could shorten but not lengthen expected asset lifespans—they were only factored into the upper estimates. To reflect the greater likelihood that water and sanitation assets will be harmed by climate hazards, the expected lifespans of on-premises water sources and septic-based sanitation systems were shortened in the upper estimates from greater than ten years to seven years. Networked water and sanitation assets were assumed to be more climate resilient[72] and thus retained expected lifespans of greater than ten years.

Table S12. Values of varied parameters across the baseline, lower, and upper estimates

Parameter	Value in baseline estimates	Value in lower estimates	Value in upper estimates
Share of full per-facility capital costs for water and sanitation assumed to be needed in facilities requiring "reduced investment"	50%	15%	85%
Discount rate	5%	8%	3%
Average expected lifespan of on-premises water sources and septic-based sanitation systems	>10 years	>10 years	7 years

One-way sensitivity analysis relative to the baseline estimates was conducted to determine the effect on estimated costs of varying each of these three parameters individually (table S13 and figure S1).

Table S13. Estimated costs with one-way sensitivity analysis for each key parameter

Modelled scenarios for sensitivity analysis	Total cost (US$)	Capital cost (US$)	Recurrent cost (US$)
Baseline estimate	7 879 083 148	3 611 389 929	4,267,693,219
Lower estimate	6,524,871,085	2,880,736,454	3,644,134,631
Upper estimate	9,558,250,725	4,790,760,379	4,767,490,346
Baseline with 8% discount rate	6,838,540,677	3,194,406,046	3,644,134,631
Baseline with 3% discount rate	8,708,671,807	3,941,181,462	4,767,490,346
Baseline with “reduced investment” at 85% of per-facility costs	8,230,016,838	3,962,323,619	4,267,693,219
Baseline with “reduced investment” at 15% of per-facility costs	7,528,149,458	3,260,456,238	4,267,693,219
Baseline with selected water and sanitation asset lifespans	8,208,438,106	3,940,744,887	4,267,693,219

Figure S1. Percentage effect of varying key parameters on capital and recurrent costs compared to the lower and upper estimates (one-way sensitivity analysis)

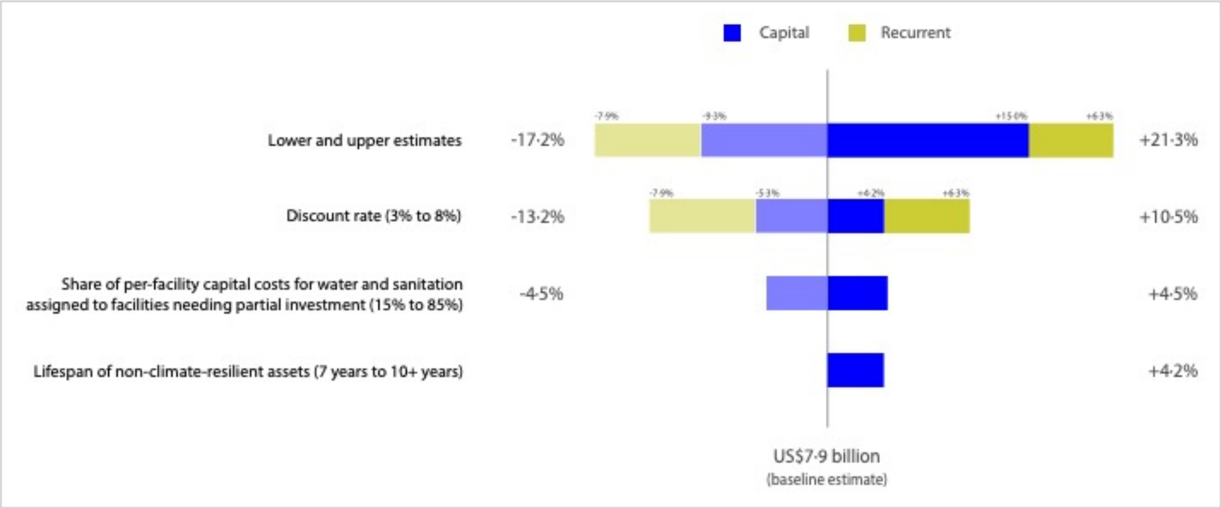

Section 9: Benchmark analysis

Information is scant regarding past and current spending levels in LDCs on WASH and waste services in health care facilities. Consequently, a funding gap analysis, which would rely on trend-based forecasts of expected spending on WASH and waste services in LCDs, was not feasible for this study. Related, no attempt was made to estimate the effectiveness or efficiency of current spending.

Instead, a basic benchmarking analysis against existing spending levels was carried out to give an indicative sense of financial feasibility for the scale-up of basic service levels in the LDCs existing public health facilities.

Secondary data were compiled from multiple sources for four relevant comparator categories of expenditure (table S14). First, data on capital health expenditure per capita, financed by both domestic public and external sources, was retrieved from WHO's Global Health Expenditure Database (GHED)[73], with estimates available for 23 LDCs. The most recent estimates available from 2015 onward were taken, which were expressed in 2018 US$.

Second, current health expenditure per capita financed by domestic public sources (GGHE-D per capita) in 2018 was also retrieved from the GHED, with estimates available for all but two LDCs. The GHED does not contain data from Somalia, and no 2018 value was reported for Yemen.

Third, WASH expenditure per capita data were reported in the UN-Water global analysis and assessment of sanitation and drinking-water (GLAAS) 2019 report for 22 LDCs. As part of GLAAS, countries self-reported spending from one of their budget years between 2017 and 2019.[74]

Finally, data on aid disbursements in 2019 for WASH in LDCs were retrieved from the OECD's Creditor Reporting System (CRS).[75] Data were downloaded from the CRS for Water Supply and Sanitation (sector 140) including all official donors, all channels, all aid types, and both Official Development Assistance (ODA) and Other Official Flows.

For all four benchmarks, population-adjusted estimates for per-capita expenditure across the LDCs were computed, in each case reflecting only the countries for which expenditure estimates were available. National population data were drawn from the World Population Prospects[76] for the years corresponding to the expenditure data. For example, the main paper alludes to the US$0·80 invested in health capital by 23 LDC governments in 2018—this amount was calculated through the following steps (an analogous process was followed for the other three spending benchmarks):

1. Retrieving from the GHED the estimates for those 23 countries' government expenditure per capita on health capital in 2018;
2. Multiplying each by their respective country's population in 2018 to compute total government expenditure on health capital;
3. Dividing that amount by the sum of the countries' populations.

Table S14. Per capita health and WASH expenditure by country (US$)

Country	Capital expenditure in health (government)*	Capital expenditure in health (external)*	Current health expenditure (government)†	WASH expenditure (government)‡	WASH expenditure (external)§
Afghanistan	··	··	2·58	1·00	2·46
Angola	··	··	36·74	··	2·01
Bangladesh	··	··	7·12	2·07	2·01
Benin	1·56	0·55	6·09	2·88	4·34
Bhutan	··	··	81·73	8·65	8·50
Burkina Faso	2·55	0·16	17·11	0·68	7·64
Burundi	0·06	0·84	5·90	··	1·70
Cambodia	0·03	0·95	19·26	··	8·99
Central African Republic	0·96	0·45	3·36	··	1·59
Chad	··	··	4·97	··	1·16
Comoros	··	··	6·04	··	3·43
Democratic Republic of the Congo	0·07	0·27	2·79	··	1·30
Djibouti	··	··	35·12	··	45·40
Eritrea	··	··	3·70	··	1·20
Ethiopia	1·31	1·07	5·66	··	2·21
Gambia (Republic of The)	0·37	0·64	6·78	0·09	0·76
Guinea	1·88	1·57	6·30	1·70	2·16
Guinea Bissau	0·07	0·43	4·88	··	3·00
Haiti	0·61	1·48	7·70	0·59	3·75
Kiribati	··	··	150·39	··	43·55
Lao People’s Democratic Republic	··	··	22·10	··	7·75
Lesotho	··	··	72·46	15·72	3·19
Liberia	0·04	1·05	11·43	··	3·58
Madagascar	··	··	7·88	0·13	1·08
Malawi	··	··	10·25	1·06	3·10
Mali	1·39	1·02	9·86	··	5·09
Mauritania	0·80	4·43	19·55	3·31	18·44
Mozambique	1·79	0·91	8·52	0·16	4·03
Myanmar	··	··	8·78	··	2·15
Nepal	··	··	14·49	12·09	3·76
Niger	0·91	0·80	10·08	0·22	5·74
Rwanda	··	··	18·35	··	3·15
São Tomé and Príncipe	1·97	9·85	55·13	··	30·15
Senegal	1·81	1·63	14·00	5·80	9·15
Sierra Leone	··	··	8·33	··	3·61
Solomon Islands	··	··	75·07	0·60	8·27
Somalia	··	··	··	··	1·13
South Sudan	0·09	1·34	2·89	0·09	3·38
Sudan	··	··	13·75	··	1·12
United Republic of Tanzania	0·14	0·34	15·81	9·65	3·98
Timor-Leste	··	··	56·82	··	3·57
Togo	0·28	0·23	7·13	0·01	1·97
Tuvalu	··	··	553·10	··	2·26
Uganda	0·25	0·83	6·83	2·61	2·80
Yemen	··	··	··	··	3·45
Zambia	0·00	0·04	29·70	2·53	5·04
**Average (population-weighted)**<br>(Number of countries included)	**0·80**<br>(23)	**0·76**<br>(23)	**10·17**<br>(44)	**3·09**<br>(22)	**3·01**<br>(46)

*Values retrieved from WHO’s Global Health Expenditure Database, taking the most recent value available from 2015 onward, all expressed in 2018 US$.
†Values retrieved from WHO’s Global Health Expenditure Database for 2018, expressed in 2018 US$.
‡Government expenditure reported in the 2018/2019 GLAAS country survey from budget years ending in 2017, 2018, or 2019. Per capita values were calculated by the authors.
§Aid disbursement values retrieved from the OECD CRS for 2019, expressed in 2018 US$. Per capita values were calculated by the authors.

References for supplementary materials

1 WHO, UNICEF. Global progress report on water, sanitation and hygiene in health care facilities: fundamentals first. Geneva: World Health Organization, 2020 https://www.who.int/publications/i/item/9789240017542 (accessed Feb 1, 2021).

2 United Nations Department of Economic and Social Affairs. Least Developed Countries (LDCs). 2020. https://www.un.org/development/desa/dpad/least-developed-country-category.html/ (accessed Feb 7, 2021).

3 United Nations Department of Economic and Social Affairs, Population Division. World Population Prospects 2019. 2019. https://population.un.org/wpp/Download/Standard/Population/ (accessed Feb 19, 2021).

4 United Nations Department of Economic and Social Affairs, Population Division. World Urbanization Prospects 2018. 2018. https://population.un.org/wup/.

5 WHO, UNICEF. Core questions and indicators for monitoring WASH in health care facilities in the Sustainable Development Goals. Geneva: World Health Organization & the United Nations Children's Fund, 2018 https://washdata.org/sites/default/files/documents/reports/2019-04/JMP-2018-core-questions-for-monitoring-WinHCF.pdf (accessed Feb 2, 2021).

6 WHO, UNICEF. WASH in health care facilities: Global Baseline Report 2019. Geneva: World Health Organization and the United Nations Children's Fund, 2019 https://www.who.int/water_sanitation_health/publications/wash-in-health-care-facilities-global-report/en/ (accessed Feb 1, 2021).

7 Hutton G, Varughese M. The Costs of Meeting the 2030 Sustainable Development Goal Targets on Drinking Water, Sanitation, and Hygiene. Washington, DC: World Bank, 2016 DOI:10.1596/K8543.

8 WHO. WHO EMRO | Health Systems | Programmes | Afghanistan. World Health Organ. Reg. Off. East. Mediterr. http://www.emro.who.int/afg/programmes/health-system-strengthening.html (accessed Feb 15, 2021).

9 USAID, US Department of Health & Human Services, CDC, US Department of State. President's Malaria Initiative Angola Malaria Operational Plan FY 2019. Washington: United States Agency for International Development https://www.pmi.gov/docs/default-source/default-document-library/malaria-operational-plans/fy19/fy-2019-angola-malaria-operational-plan.pdf?sfvrsn=3.

10 Government of the People's Republic of Bangladesh Ministry of Health and Family Welfare. Health Bulletin 2019. Mohakhali: Government of the People's Republic of Bangladesh Ministry of Health and Family Welfare Division of Management Information System, 2020 https://dghs.gov.bd/images/docs/Publicaations/Health%20Bulletin%202019%20Print%20Version%20(2)-Final.pdf.

11 Ministére de la Santè République du Bénin. Annuaire Des Statistiques Sanitaires 2019. Cotonou: Ministére de la Santè République du Bénin, 2020 https://sante.gouv.bj/MS_console/recherche_avancee.

12 Ministry of Health Royal Government of Bhutan. Annual Health Bulletin 2020. Thimphu: Ministry of Health Royal Government of Bhutan Policy and Planning Division, 2020 http://www.moh.gov.bt/wp-content/uploads/ict-files/2017/06/health-bulletin-Website_Final.pdf.

13 Institut national de la statistique et de la démographie. Annuaire Statistique 2018. Ouagadougou: Ministère de l'économie des finances et du développement Institut national de la statistique et de la démographie, 2019 http://www.insd.bf/contenu/pub_periodiques/annuaires_stat/Annuaires_stat_nationaux_BF/Annuaire_Statistique_National_2018.pdf.

14 World Bank, UNICEF Burundi. Burundi Delivering Health Services Under Fiscal Stress. Washington: World Bank, 2017 https://www.unicef.org/esaro/PER-of-Health-in-Burundi-(2017).pdf.

15 Ministry of Health Kingdom of Cambodia. Health strategic plan 2016-2020. Phnom Penh: Ministry of Health Kingdom of Cambodia Department of Planning & Health Information, 2016 http://hismohcambodia.org/public/fileupload/carousel/HSP3-(2016-2020).pdf.

16 WHO. HeRAMS - République Centrafricaine Dashboard. Health Resour. Serv. Availab. Monit. Syst. 2021. https://herams.org/ (accessed Feb 1, 2021).

17 Ministère de la Santé Publique et de la Solidarité Nationale du Tchad, WHO, UNICEF, et al. Plan National De Developpement Sanitaire (PNDS3: 2018-2021). N'Djamena: Ministère de la Santé Publique et de la Solidarité Nationale du Tchad, 2018 https://sante-tchad.org/documents-strategiques/.

18 World Bank. Project Appraisal Document on a Proposed Grant in the Amount of SDR 10.9 Million (US $15.0 Million Equivalent) and a Proposed Credit in the Amount of EUR 13.4 Million (US$15.0 Million Equivalent) to the Union of Comoros for the Comprehensive Approach to Health Systems Strengthening Project. Washington: World Bank, 2019 http://documents1.worldbank.org/curated/en/988861560788593578/pdf/Comoros-Comprehensive-Approach-to-Health-System-Strengthening-Project.pdf.

19 Brunner B, Combet V, Callahan S, et al. The Role of the Private Sector in Improving the Performance of the Health System in the Democratic Republic of Congo. Bethesda: Abt Associates Inc., 2018 http://documents1.worldbank.org/curated/en/487571539958646859/pdf/131045-REVISED-23-1-2019-10-49-58-WBDRCPSAEnglishWEB.pdf.

20 République de Djibouti Ministére de la Santé. Projet De Riposte A La Pandemic De COVID-19 (P173807)-Cadre de Gestion Environmentale ET Socialepour La Lutte Contre La COVID-19. Djibouti: République de Djibouti Ministére de la Santé, 2020 https://sante.gouv.dj/storage/publications/October2020/OReIPaoUzz2tVQXGyZae.pdf.

21 State of Eritrea Ministry of Health. National Action Plan for Health Security (NAPHS) 2017-2021. Asmara: State of Eritrea Ministry of Health, 2017 https://extranet.who.int/sph/sites/default/files/document-library/document/Final%20NAPHS%20Eritrea%20Document%2018%20August%202017.pdf.

22 Federal Ministry of Health Ethiopia. National Cancer Control Plan 2016-2020. Addis Ababa: Federal Ministry of Health Ethiopia, 2015 https://www.iccp-portal.org/sites/default/files/plans/NCCP%20Ethiopia%20Final%20261015.pdf.

23 Sine J, Pascal Saint-Firmin P, Williamson T. Assessment of the health System in the Gambia: Overview, Medical Products, Health Financing, and Governance Components. Washington: Palladium & Health Policy Plus, 2019 http://www.healthpolicyplus.com/ns/pubs/17372-17674_GambiaHealthSystemAssessment.pdf.

24 République de Guinée Ministere de la Santé. Plan National De Developpement Sanitaire (PNDS) 2015-2024. Conakry: République de Guinée Ministere de la Santé, 2015 https://www.prb.org/wp-content/uploads/2018/05/Plan-National-de-De%CC%81veloppement-Sanitaire-2015-2024.-Guine%CC%81e.pdf.

25 Kitson N. Informal payments in the public health sector in Guinea-Bissau. London: Overseas Development Institute, 2019 https://www.odi.org/sites/odi.org.uk/files/resource-documents/12949.pdf.

26 Institut Haïtien de l'Enfance, ICF International. Évaluation de la Prestation des Services de Soins de Santé (EPSSS), Haïti, 2013. Rockville: Institut Haïtien de l'Enfance & ICF International, 2014 http://mspp.gouv.ht/site/downloads/EPSSS.pdf (accessed Feb 17, 2021).

27 World Bank Group, Australian Aid, New Zealand Ministry of Foreign affairs & Trade. Kiribati Health Financing System Assessment Spend Better. Washington: World Bank Group, 2018 http://documents1.worldbank.org/curated/en/211441528787830345/pdf/Kiribati-HFSA.pdf.

28 Ministry of Health Lao People's Democratic Republic, WHO. Overview of Lao Health System Development 2009-2017. Manila: World Health Organization Regional Office for the Western Pacific, 2018 https://apps.who.int/iris/bitstream/handle/10665/274721/9789290618621-eng.pdf.

29 Ministry of Finance Government of the Kingdom of Lesotho, UNICEF. Lesotho Health Budget Brief Fiscal Year 2018-19. Maseru: United Nations Children's Fund, 2018 https://lesotho.un.org/sites/default/files/2019-10/Lesotho%20Health%20Budget%20Brief%202018-19.pdf.

30 Ministry of Health Government of Liberia. Investment Plan for Building a Resilient Health System, Liberia 2015-2021. Monrovia: Ministry of Health Government of Liberia, 2015 http://pubdocs.worldbank.org/en/865131479921763514/Liberia-Investment-Plan-Final-May-15.pdf.

31 Ministere de la Sante Publique Madagascar. Annuaire des Statistiques du Secteur Sante de Madagascar Annee 2016. Antananarivo: Ministere de la Sante Publique Madagascar Direction des Estudes et de la Planification Sevice des Statistiques Sanitaires et Demographiques, 2016 https://www.instat.mg/wp-content/uploads/2019/03/MINSANP_Annuaire-Stat_2016.pdf.

32 The Health Care System. Minist. Health Popul. Repub. Malawi. https://www.health.gov.mw/index.php/2016-01-06-19-58-23/national-aids (accessed Feb 1, 2021).

33 World Bank. Mali - Accelerating Progress Towards Universal Health Coverage (P165534): Combined Project Information Documents / Integrated Safeguards Datasheet (PID/ISDS). Washington: World Bank, 2018 http://documents1.worldbank.org/curated/en/929821547659438840/pdf/Project-Information-Document-Integrated-Safeguards-Data-Sheet-Mali-Accelerating-Progress-Towards-Universal-Health-Coverage-P165534.pdf.

34 World Bank. International Development Association Project Appraisal Document on a Proposed Grant in the Amount of SDR 12.6 Million (US $17.0 Million Equivalent) to the Islamic Republic of Mauritania for a Health System Support Project (INAYA). Washington: World Bank, 2017 http://documents1.worldbank.org/curated/en/922961495418418570/pdf/Mauritania-PAD-05022017.pdf.

35 USAID. Overview of Private Actors in the Mozambican Health System and Rapid Assessment of the Supply Chain. Washington: United States Agency for International Development, 2019 https://thinkwell.global/wp-content/uploads/2020/02/AID-656-C-17-00002-Private-Sector-Final-Revised-Report.pdf.

36 Eurocham Myanmar, CCI France Myanmar. Healthcare Guide 2018. Yangon: European Chamber of Commerce in Myanmar, 2017 https://www.ccifrance-myanmar.org/sites/ccifrance-myanmar.org/files/resources-documents/healthcare_guide_2018.pdf.

37 Government of Nepal Ministry of Health and Population. Number of Health Facilities in Nepal – Health Emergency Operation Center. 2017. https://heoc.mohp.gov.np/service/number-of-health-facilities-in-nepal/ (accessed Dec 11, 2020).

38 Republique du Niger Ministere du Plan. Annuaire Statistique 2013-2017 Edition 2018. Niamey: Republique du Niger Ministere du Plan Institut National de la Statistique, 2018 https://www.stat-niger.org/wp-content/uploads/2020/06/Annuaire_Statistique_2013-2017.pdf.

39 Republic of Rwanda Ministry of Health. Rwanda Health Sector Performance Report 2017-2019. Kigali: Republic of Rwanda Ministry of Health, 2020 https://www.moh.gov.rw/fileadmin/user_upload/Moh/Publications/Reports/FINAL_Annual_Report_2017-2019_02062020.pdf.

40 República Democrática de São Tomé e Principe Ministério da Saúde. Estratégia integrada de Saúde Reprodutiva, Materna, Neonatal, Infantil e do Adolescente e Nutrição 2019-2023. São Tomé: República Democrática de São Tomé e Principe Ministério da Saúde, 2018 http://ms.gov.st/wp-content/uploads/2020/06/PLANO-SAUDE-REPRODUTIVA-2019-2023.pdf.

41 République du Sénégal Ministre de la Santé et de l'Action sociale. Annuaire Statistiques Sanitaires et Sociales du Sénégal 2016. Dakar: République du Sénégal Ministre de la Santé et de l'Action sociale, 2016 http://www.sante.gouv.sn/sites/default/files/Annuaire%20statistiques%20sanitaires%20et%20sociales%202016.pdf (accessed Feb 17, 2021).

42 Ministry of Health and Sanitation Sierra Leone. National Health Sector Strategic Plan 2017-2021. Freetown: Ministry of Health and Sanitation Sierra Leone, 2017 https://extranet.who.int/countryplanningcycles/sites/default/files/planning_cycle_repository/sierra_leone/sierra_leone_nhssp_2017-21_final_sept2017.pdf.

43 WHO. HeRAMS - Somalia Dashboard. Health Resour. Serv. Availab. Monit. Syst. 2021. https://herams.org/project/41 (accessed Feb 1, 2021).

44 Republic of South Sudan Ministry of Health. 9th Annual Report 2019 Health Management Information System (HMIS). Juba: Republic of South Sudan Ministry of Health, 2020 https://southsudanhealth.info/PublicData/Library/HMIS_Annual_Report_/Final%202019%20Annual%20Report%20Republic%20of%20South%20Sudan%20Ministry%20of%20Health%20HMIS.pdf.

45 Federal Ministry of Health Sudan. Annual Health Statistical Report 2019. Khartoum: Sudan Health Observatory, 2019 http://sho.gov.sd/controller/knowledge_hub.php?&page=2&mid=110&lid=1#.

46 The United Republic of Tanzania National Bureau of Statistics. 2019 Tanzania in Figures. Dodoma: The United Republic of Tanzania National Bureau of Statistics, 2020 https://www.nbs.go.tz/nbs/takwimu/references/Tanzania_in_Figures_2019.pdf.

47 Direção Geral de Estatística. Timor-Leste In Figures 2018. Dili: Direção Geral de Estatística de Timor-Leste, 2019 https://www.statistics.gov.tl/wp-content/uploads/2020/03/Timor-Leste-In-Number-2018.pdf.

48 Republique Togolaise Ministere de la Sante et de la Protecion. Annuaire des Statistiques Sanitaires du Togo Année 2016. Lomé: Republique Togolaise Ministere de la Sante et de la Protecion Division de l'Information Sanitaire, 2017 https://www.afro.who.int/sites/default/files/2017-10/Principaux%20indicateurs%20de%20sant%C3%A9%202016.pdf.

49 WHO. Tuvalu-WHO Country Cooperation Strategy 2018-2022. Manila: World Health Organization, 2017 https://iris.wpro.who.int/bitstream/handle/10665.1/13958/WPRO-2017-DPM-024-tuv-eng.pdf.

50 The Republic of Uganda Ministry of Health. National Health Facility Master List 2018: A Complete List of All Health Facilities in Uganda. Kampala: The Republic of Uganda Ministry of Health Division of Health Information, 2018 http://library.health.go.ug/sites/default/files/resources/National%20Health%20Facility%20Master%20List%202018_0.pdf.

51 Miyajima T, Kazem AMI. Yemen: Immediate Priorities for Post-Conflict Recovery of the Health Sector. Washington: World Bank Group, 2017 http://documents1.worldbank.org/curated/en/349331508408515508/pdf/120530-WP-P159636-PUBLIC-Yemen-Health-Policy-Note-Input-to-PN-RA-edits.pdf.

52 Ministry of Health Zambia. Master Facility List. https://mfl.moh.gov.zm/list?name=&HMIS_Code=&service_category=&service=&facility_type=13&ownership=&operation_status= (accessed Feb 1, 2021).

53 Feeny S, de Silva A. Measuring absorptive capacity constraints to foreign aid. Econ Model 2012; 29: 725–33.

54 Nyarko KB, Dwumfour-Asare B, Moriarty P, Appiah-Effah E, Obuobisa-Darko A. Life-cycle costs approach for WASH services that last: Life-cycle costs in Ghana Briefing Note 6: Functionality of rural water systems in Ghana. Accra: IRC International Water and Sanitation Centre, 2012 https://www.ircwash.org/sites/default/files/briefing_note_6_functionality_of_rural_water_systems_in_ghana.pdf (accessed Jan 26, 2021).

55 Engineering For Change. RWC Rainwater Harvesting System. https://www.engineeringforchange.org/solutions/product/rwc-rainwater-harvesting-system/ (accessed Jan 25, 2021).

56 Martinson DB. Improving the Viability of Roofwater Harvesting in Low-Income Countries. 2007; published online Oct. https://warwick.ac.uk/fac/sci/eng/research/grouplist/structural/dtu/pubs/reviewed/rwh/dbm_thesis/dbm_thesis.pdf (accessed Jan 26, 2021).

57 Clasen T, Haller L, Walker D, Bartram J, Cairncross S. Cost-effectiveness of water quality interventions for preventing diarrhoeal disease in developing countries. J Water Health 2007; 5: 599–608.

58 Eco-San Toilet. ANSI Sanit. https://sanitation.ansi.org/EcoSanToilet (accessed Jan 25, 2021).

59 Engineering for Change. Crestanks WonderLoo | Engineering For Change. https://www.engineeringforchange.org/solutions/product/crestanks-wonder-loo/ (accessed Jan 25, 2021).

60 Engineering for Change. Loowatt Toilet. https://www.engineeringforchange.org/solutions/product/loowatt-toilet/ (accessed Jan 25, 2021).

61 CNA e-Tool Estimated Useful Life Table. US Dep. Hous. Urban Dev. https://www.hud.gov/sites/documents/EUL_FOR_CNA_E_TOOL.PDF (accessed Jan 25, 2021).

62 UNICEF. Handwashing Stations and Supplies for the COVID-19 Response. Washington: United Nations Children's Fund, 2020 https://www.unicef.org/media/75706/file/Handwashing%20Facility%20Worksheet.pdf (accessed Jan 25, 2021).

63 The Mark 8a. Montfort Med. Waste. https://mw-incinerator.info/en/303_Mark_8a.html (accessed Jan 25, 2021).

64 Biomedical Engineering Advisory Group. Life Span of Biomedical Devices - Guidance Paper. Clinical Engineering Division Global, 2004 http://cedglobal.org/download/Life%20Span%20of%20Biomedical%20Devices%20-%20Guidance%20Paper%20Final.pdf (accessed Jan 25, 2021).

65 FDA. 510(k) Summary. 2013; published online Nov. https://www.accessdata.fda.gov/cdrh_docs/pdf13/K133031.pdf (accessed Jan 26, 2021).

66 Engineering For Change. T4T Mak IV Incinerator. https://www.engineeringforchange.org/solutions/product/t4t-mak-iv-incinerator/ (accessed Jan 25, 2021).

67 Medical Incinerator. Solid Waste Incinerator. https://www.medical-incinerator.com/manufacturer/solid-waste-incinerator/ (accessed Jan 25, 2021).

68 Anna Vassall, Sedona Sweeney, Jim Kahn, et al. Reference case for estimating the costs of global health services and Interventions. Global Health Costing Consortium, 2017 https://researchonline.lshtm.ac.uk/id/eprint/4653001.

69 Bertram MY, Lauer JA, Stenberg K, Edejer TTT. Methods for the Economic Evaluation of Health Care Interventions for Priority Setting in the Health System: An Update From WHO CHOICE. Int J Health Policy Manag 2021; : 1.

70 Belli P, Anderson J, Barnum H, Dixon J, Tan J-P. Handbook on Economic Analysis of Investment Operations. Washington, DC: Operational Core Services Network Learning and Leadership Center, World Bank, 1998 DOI:10.1596/0-8213-4850-7.

71 Turner HC, Lauer JA, Tran BX, Teerawattananon Y, Jit M. Adjusting for Inflation and Currency Changes Within Health Economic Studies. Value Health 2019; 22: 1026–32.

72 WHO. WHO Guidance for Climate Resilient and Environmentally Sustainable Health Care Facilities. Geneva: World Health Organization, 2020 https://www.who.int/publications/i/item/9789240012226 (accessed March 12, 2021).

73 WHO. Global Health Expenditure Database. World Health Organ. 2021. https://apps.who.int/nha/database (accessed Feb 17, 2021).

74 UN Water, WHO. National systems to support drinking-water, sanitation and hygiene: global status report 2019. UN-Water global analysis and assessment of sanitation and drinking-water (GLAAS) 2019 report. Geneva: World Health Organization, 2019 https://apps.who.int/iris/bitstream/handle/10665/326444/9789241516297-eng.pdf?ua=1 (accessed Nov 18, 2020).

75 OECD. Creditor reporting system (CRS). Paris: Organisation for Economic Co-operation and Development, 2021 https://stats.oecd.org/Index.aspx?DataSetCode=crs1 (accessed Feb 18, 2021).

76 United Nations Department of Economic and Social Affairs. World Population Prospects 2019. 2019. https://population.un.org/wpp/ (accessed May 17, 2021).
